# Construction of a prognostic model with histone modification-related genes and identification of potential drugs in pancreatic cancer

**DOI:** 10.1186/s12935-021-01928-6

**Published:** 2021-06-05

**Authors:** Yuan Chen, Ruiyuan Xu, Rexiati Ruze, Jinshou Yang, Huanyu Wang, Jianlu Song, Lei You, Chengcheng Wang, Yupei Zhao

**Affiliations:** Department of General Surgery, State Key Laboratory of Complex Severe and Rare Diseases, Peking Union Medical College Hospital, Chinese Academy of Medical Sciences, Peking Union Medical College, Beijing, 100023 People’s Republic of China

**Keywords:** Histone modification, Prognostic model, CBX8, CENPT, DPY30, PADI1, Potential drugs, Pancreatic cancer

## Abstract

**Background:**

Pancreatic cancer (PC) is a highly fatal and aggressive disease with its incidence and mortality quite discouraging. An effective prediction model is urgently needed for the accurate assessment of patients’ prognosis to assist clinical decision-making.

**Methods:**

Gene expression data and clinicopathological data of the samples were acquired from The Cancer Genome Atlas (TCGA), Genotype-Tissue Expression (GTEx), and Gene Expression Omnibus (GEO) databases. Differential expressed genes (DEGs) analysis, univariate Cox regression analysis, least absolute shrinkage and selection operator (LASSO) regression analysis, random forest screening and multivariate Cox regression analysis were applied to construct the risk signature. The effectiveness and independence of the model were validated by time-dependent receiver operating characteristic (ROC) curve, Kaplan–Meier (KM) survival analysis and survival point graph in training set, test set, TCGA entire set and GSE57495 set. The validity of the core gene was verified by immunohistochemistry and our own independent cohort. Meanwhile, functional enrichment analysis of DEGs between the high and low risk groups revealed the potential biological pathways. Finally, CMap database and drug sensitivity assay were utilized to identify potential small molecular drugs as the risk model-related treatments for PC patients.

**Results:**

Four histone modification-related genes were identified to establish the risk signature, including CBX8, CENPT, DPY30 and PADI1. The predictive performance of risk signature was validated in training set, test set, TCGA entire set and GSE57495 set, with the areas under ROC curve (AUCs) for 3-year survival were 0.773, 0.729, 0.775 and 0.770 respectively. Furthermore, KM survival analysis, univariate and multivariate Cox regression analysis proved it as an independent prognostic factor. Mechanically, functional enrichment analysis showed that the poor prognosis of high-risk population was related to the metabolic disorders caused by inadequate insulin secretion, which was fueled by neuroendocrine aberration. Lastly, a cluster of small molecule drugs were identified with significant potentiality in treating PC patients.

**Conclusions:**

Based on a histone modification-related gene signature, our model can serve as a reliable prognosis assessment tool and help to optimize the treatment for PC patients. Meanwhile, a cluster of small molecule drugs were also identified with significant potentiality in treating PC patients.

**Supplementary Information:**

The online version contains supplementary material available at 10.1186/s12935-021-01928-6.

## Background

Pancreatic cancer is one of the most lethal malignancies, with a 5-year survival rate of only 10% in the United States [1]. According to the latest epidemiological data, it is reported that there are 495,773 new cases worldwide in 2020, and approximately 466,003 will die of this disease, ranking 14th and 7th among all cancers respectively [2]. In addition to the malignant characteristics of the PC itself and the lack of early screening methods, the disappointing prognosis of this disease is largely attributable to the lack of effective risk prediction models. Although advanced surgical techniques, targeted drugs, radiotherapy and chemotherapy drugs have been applied, the survival benefits of these treatments are questionable and side effects occur to varying degrees for each individual [3, 4]. The treatment of PC should be individualized and systematic, which can ensure a maximum gain and minimum risk. Therefore, an effective prediction model is urgently needed for the accurate assessment of patients’ prognosis to assist clinical decision-making. In this context, doctors can decide whether a more aggressive treatment regimen is needed, and an efficacious treatment with balanced benefits and side effects can be selected.

Traditional risk stratification systems, such as AJCC TNM staging, has been proved to have nondiscriminatory predictive efficacy for PC [5, 6]. However, more predictive models are required to evaluate the prognosis of PC in multiple dimensions. With the large-scale application of next-generation sequencing and microarray technology in disease research, the use of specific gene expression levels and mutation status to assess disease prognosis has become increasingly accessible [7, 8]. Actually, a number of studies have demonstrated that specific gene expression or mutant characteristics can effectively predict the prognosis of tumors [9, 10]. Concomitantly, the establishment of prognostic models for PC has also been extensively reported, including whole-gene prediction model [11–13], lncRNA-related prediction model [14], immune gene-related prediction model [15], and ceRNA-related model [16], etc. At the same time, the pivotal role of histone modification in the progression of PC is gradually revealed in recent years. Histone modifications, mainly involving acetylation, methylation, phosphorylation and ubiquitylation, represent a versatile set of epigenetic marks that regulate multiple dynamic cellular processes [17]. Under certain conditions, aberrant histone modifications of cancer-related genes may lead to abnormal activation or silencing of these genes, leading to the occurrence and progression of PC. Moreover, the changes of histone modifiers tend to regulate the histone modification of a list of genes rather than targeting a specific gene. Therefore, it may lead to a series of gene expression changes and produce a large-scale genetic remodeling in cells, which further demonstrates the pivotal role of histone modification in the development of PC. For example, the transcription factor FOXA1 promotes PC progression by driving large-scale enhancer reprogramming through H3K27ac (acetylation of lysine 27 of histone 3) modulation [18]. Histone lysine methyltransferases enhancer of zeste homolog 2 (EZH2) has been found to act as both an oncogene and a tumor suppressor, since it maintains, rather than specifies, the transcriptional repression state of thousands of target genes [19]. In view of the proposed concept of histone code and the critical role of histone modification in the progression of PC [20], we consider a prognosis signature based on histone modification-related genes to better predict the prognosis of PC patients and optimize the clinical decision-making.

In this study, we reported a risk signature model based on genes associated with histone modification to predict the prognosis of PC patients. Four histone modification-related genes were identified to construct the risk signature, which was proved to be an independent risk factor and was validated in the training set, testing set, entire set and GSE57495 set. Furthermore, functional enrichment analysis showed that the poor prognosis of high-risk population was related to the metabolic disorders caused by inadequate insulin secretion, which was fueled by neuroendocrine abnormality. Finally, CMap database and drug sensitivity assay were utilized to identify potential drugs as the risk model-related treatments for PC patients.

## Methods

### Datasets sources and processing

Histone modification-related genes were extracted from the in Gene Set Enrichment Analysis (GSEA) website (https://www.gsea-msigdb.org/gsea/index.jsp; ≤ Sep 1, 2020). Gene expression data was downloaded from UCSC XENA website (https://xenabrowser.net/datapages/; ≤ Sep 1, 2020), which included 167 normal tissues and 179 tumor tissues of pancreas. For all tumor samples in subsequent analysis gene expression data, clinical characteristics, and survival information of the patients were downloaded from The Cancer Genome Atlas (TCGA) dataset (https://portal.gdc.cancer.gov/; ≤ Sep 1, 2020). We matched the sequencing data with the clinical information and removed samples with insufficient information of status, life span, age, gender, TNM, AJCC stage and grade. Finally, 171 cases with corresponding tumor tissues and clinical information were included in the study (Table 1, detailed in Additional file 1: Table S1). Transcripts Per Kilobase Million (TPM) for each gene were calculated, and log2(TPM + 0.01) was used in subsequent analyses. The patients (n = 171) were further randomly assigned to a training set and a testing set by a ratio of 7:3. Gene IDs from gene expression data were converted to gene symbol by using a GFF3 file, which was downloaded from GENCODE (https://www.gencodegenes.org/).

**Table 1:** Clinical and pathologic information of training set, test set and entire set

Character	Training set		Test set		Entire set	
	Number	%	Number	%	Number	%
Risk score						
Median	0.994441		0.843157		0.925682	
Range	0.074169–5.458149		0.186167–2.964505		0.074169–5.458149	
Age						
Median	65		64		65	
Range	35–85		39–88		35–88	
Gender						
Male	65	55.08	28	52.83	93	54.39
Female	53	44.92	25	47.17	78	45.61
AJCC_stage						
I + II	112	94.92	52	98.11	164	95.91
III + IV	6	5.08	1	1.89	7	4.09
Grade						
G1 + G2	88	74.58	33	62.26	121	70.76
G3 + G4	30	25.42	20	37.74	50	29.24
T						
T1 + T2	20	16.95	10	18.87	30	17.54
T3 + T4	98	83.05	43	81.13	141	82.46
N						
N0 + NX	37	31.36	13	24.53	50	29.24
N1	81	68.64	40	75.47	121	70.76
M						
M0 + MX	115	97.46	52	98.11	167	97.66
M1	3	2.54	1	1.89	4	2.34

Meanwhile, one microarray dataset GSE57495 which included 63 PC patients with corresponding clinical information (Table 2, detailed in Additional file 2: Table S2) was downloaded from GEO (http://www.ncbi.nlm.nih.gov/geo/) for external validation. It was performed on GPL15048 platform. Expression values were calculated using the robust multi-array average (RMA) algorithm. The normalized expression matrix of microarray data can be directly download from the dataset. The probes were annotated by using the corresponding annotation files from the dataset as well. The principal component analysis (PCA) was used to detect whether the dataset had the batch effect. The “sva” R package was used to eliminate the batch effect [21].

**Table 2:** Clinical information of GSE57495 dataset

Character	Total (N = 63)		High risk (N = 31)		Low risk (N = 32)	
	Number	%	Number	%	Number	%
Risk score						
Median	1.202208		1.202208		1.173695	
Range	0.213675–3.129482		1.230773–3.129482		0.213675–1.202208	
OS						
Median	21.1		21.1		21.7	
Range	2.9–79.8		3.8–49.5		2.9–79.8	
Status						
Alive	21	33.33	7	22.58	14	43.75
Dead	42	66.67	24	77.42	18	56.25
AJCC_stage						
I	13	20.63	4	12.90	9	28.13
II	50	79.37	27	87.10	23	71.87

### Construction and assessment of a risk signature associated with survival of PC patients

To screen genes for constructing risk signature, DEGs between tumor- and non-tumor tissues were identified using edgeR package in R [22]. |log2FC| > 1.5 and false discovery rate (FDR) < 0.001 were set as the cutoffs for the DEGs. And those genes with their P values < 1 × 10^–100^ and |logFC| > 2 were labelled in the volcano plot by ggrepel package in R. Meanwhile, univariate Cox regression models were performed to select genes that were associated with overall survival (OS) of PC patients in the training set. P < 0.05 was considered statistically significant. Finally, the union of the above two gene sets was selected as candidate genes. LASSO regression and random forest screening were used to screen out the optimal gene combination for constructing the risk signature. Then multivariate Cox regression model was performed to further identify the selected genes using step function in R, and the risk signature was established according to the regression coefficient-weighted pseudogene expression. The risk score was calculated as follows: Risk score = (expr_gene1_ × Coef_gene1_) + (expr_gene2_ × Coef_gene2_) + $\cdots$ + (expr_genen_ × Coef_genen_). Based on the risk score formula, PC patients were divided into high risk and low risk groups. The Kaplan–Meier (K–M) curve, time-dependent receiver operating characteristic (ROC) curve and survival point diagram were utilized to assess the efficiency and independence of the risk signature in training set, testing set, entire TCGA set, and GSE57495 set, respectively. Heatmaps of the differential expression of the four predicted genes was also used to confirm this result.

In addition, to better predict the 1-, 2-, and 3-year survival of PC patients, the risk score and clinicopathological factors were incorporated to establish a nomogram, which was based on the results of the univariate and multivariate analysis by using the ‘rms’ package in R language. The time-dependent ROC curve and calibration curve of 1-, 2-, and 3-year survival were used to evaluate the efficiency of the nomogram.

Then we obtained the mutation information of 4 genes from the cBioPortal database (http://www.cbioportal.org/). The protein interaction network of the four genes was also obtained from STRING database (https://string-db.org/) and verified with the ‘corrplot’ package in the TCGA dataset. Meanwhile, univariate Cox regression analyses, multivariate Cox regression analyses and hierarchical analysis were conducted to evaluate the independency of the risk signature.

### Clinical specimens

The specimens with corresponding clinical and pathologic data of 81 patients who underwent pancreatic cancer surgery in the Peking Union Medical College Hospital and Cancer Hospital Chinese Academy of Medical Sciences between 2014 and 2019 were collected. OS was calculated from the initial surgery to the date of death or the last follow-up. This project was approved by the Ethics Committee of Peking Union Medical College Hospital and Cancer Hospital Chinese Academy of Medical Sciences. Written informed consent were obtained from all the patients enrolled in this study.

### Immunohistochemistry

Tissue paraffin were cut into 4 μm-thick sections and then deparaffinized with xylenes and rehydrated, submerged into EDTA buffer (pH 9.0) antigen retrieval buffer and microwaved for antigenic retrieval. They were treated with peroxidase blocking solution for 30 min and then were treated with normal goat serum for 30 min. Consequently, the sections were incubated with rabbit polyclonal antiDPY30 antibody (1:300) (Sigma-Aldrich, St. Louis, USA) for 2 h and then incubated with horseradish peroxidase-labeled goat anti-rabbit IgG complex (OriGene Technologies, Inc., Beijing, China) at for 30 min at room temperature. Bound antibodies were detected using diaminobenzidine. Finally, the slides were counterstained with haematoxylin. The immunohistochemical evaluation was performed by two experienced pathologists who had no knowledge of the clinical status of the patients according to the intensity of staining and the percentage of stained tumor cells. Digital images were taken and processed using Leica Microsystems and Leica Application Suite V4 (Leica, Solms, Germany).

### Differential gene analysis, co-expression network construction and functional enrichments analysis between high and low risk groups

We also used the edgeR package to perform differential genetic analysis of patients between high and low risk groups in entire set [22], |log2FC| > 2 and FDR < 0.001 were considered statistically significant. 50 genes with the most significant differences were shown and each patient’s clinical information was labeled at the top of the heatmap. Afterwards, the co-expression network was constructed and visualized with STRING database and Cytoscape. We set the minimum required interaction score to be high confidence (0.700) and hid disconnected nodes in the network, therefore not all genes were represented. In order to elucidate the molecular mechanisms of the prognostic differences between high and low risk groups, the ALL ontology of the DEGs was analyzed by Gene Ontology (GO) [23], while pathway enrichment was analyzed by the Kyoto Encyclopedia of Genes and Genomes (KEGG) [24]. In addition, in order to reduce the bias, gene set enrichment analysis (GSEA) was performed on all genes [25], whether or not they reached the difference threshold. R package clusterProfiler was used in this process [26]. P < 0.05 was considered statistically significant.

### Candidate drugs prediction by CMap database

CMap database (https://clue.io/) was applied to identify novel candidate drugs for the risk signature, which compare the similarity of the differentially expressed gene profiles with the expression profiles of various small molecular compound treated cell lines. Score was used to evaluate the degree of similarity, which ranged from − 100 to 100. The results were ordered from low to high. The lower the value, the more opposite the gene expression profile treated with the small molecule drug was to the difference gene profile of the high and low risk group, which indicated it had a potential therapeutic effect for high-risk patients and low-risk patients respectively.

### Cell culture

The pancreatic cancer cell lines PANC-1, MIACaPa-2, BxPC-3 and CFPAC-1 were purchased from the American Type Culture Collection (ATCC) and tested for mycoplasma every two months. PANC-1 and MIACaPa-2 cells were cultured in high glucose Dulbecco’s modified Eagle’s medium (DMEM; CORNING, Manassas, USA), BxPC-3 cell line was cultured in RPMI-1640 medium (CORNING, Manassas, USA), and CFPAC-1 cell line was cultured in Iscove’s Modified Dulbecco Medium (IMDM; CORNING, Manassas, USA). All medium was supplemented with 10% fetal bovine serum (FBS; HyClone; GE Healthcare Life Sciences, Logan, UT, USA). Cells were maintained at 37 °C with 5% CO_2_ in a humidified incubator.

### Drug sensitivity assay

Drugs were purchased from MedChemExpress (New Jersey, USA), dissolved in dimethyl sulfoxide (DMSO), and stock solutions stored at − 80 °C. Pancreatic cancer cell lines were seeded at a density of 3000 cells per well in 96-well flat-bottomed culture dishes. After overnight incubation, each drug was added at the indicated concentrations and incubated for three days; assays were performed in triplicate. Samples were evaluated for relative cell number using Cell Counting Kit-8 (CCK-8) reagent (Dojindo, Tokyo, Japan). Results were quantified using a fluorescence microplate reader by measuring fluorescence of CCK-8 at an excitation wavelength of 450 nm with fluorescence emission at 630 nm. Results were analyzed using GraphPad Prism 8.0 to determine the IC50 for each drug.

### Statistical analysis

The samples of tumor tissues in TCGA set were randomly divided into two groups using “sample” function of R software. All the statistical analyses and visualization, including PCA analysis, DEGs analysis, univariate and multivariate Cox regression analysis, LASSO regression and random forest screening, ROC curve analysis and K–M survival analyses were performed using Rstudio (version 4.0.2). All statistical tests were two-sided. P < 0.05 was considered as statistically significant unless otherwise noted.

## Results

### Four histone modification-related genes were screened out for constructing a risk signature

**Fig. 1:**
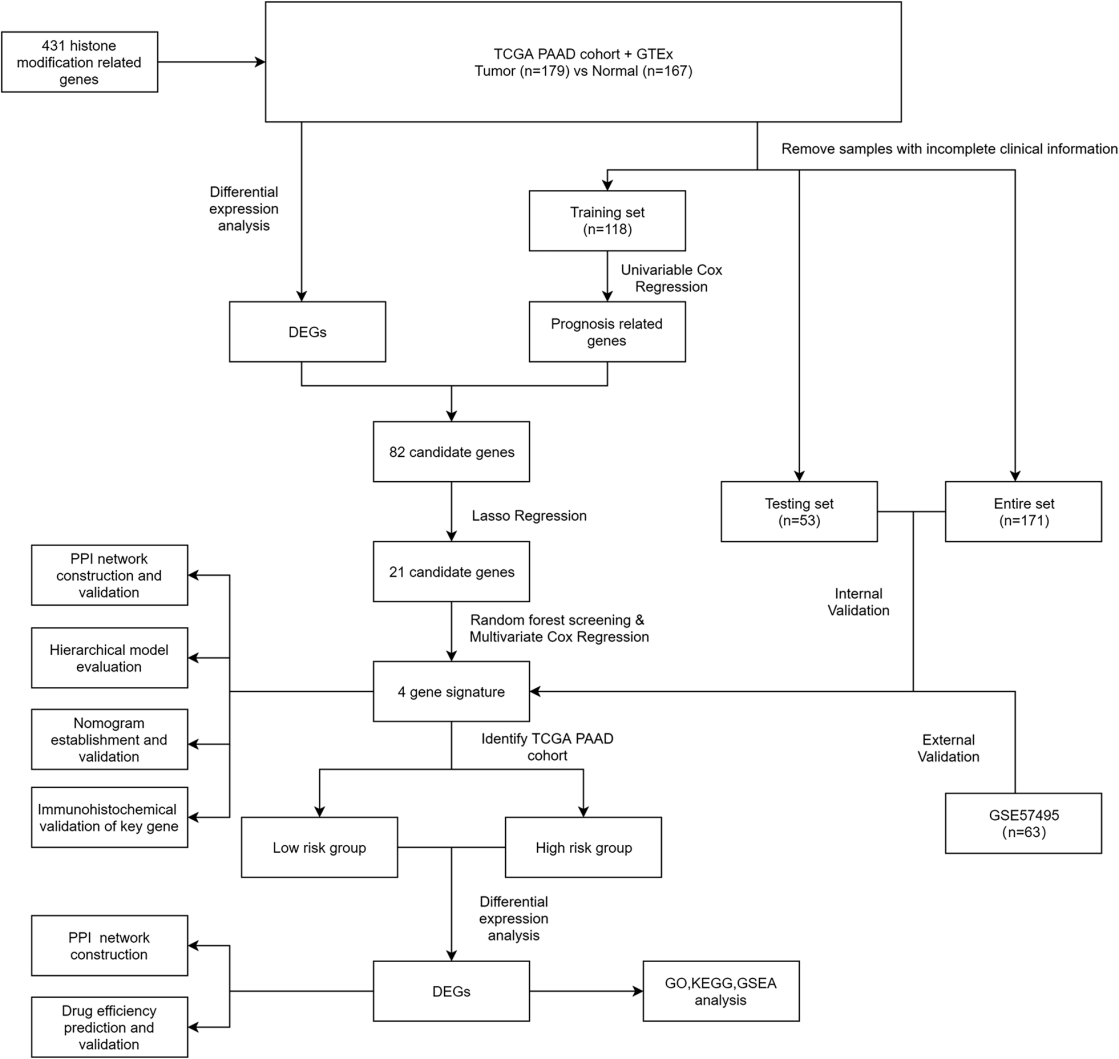
Flowchart of the whole study

**Fig. 2:**
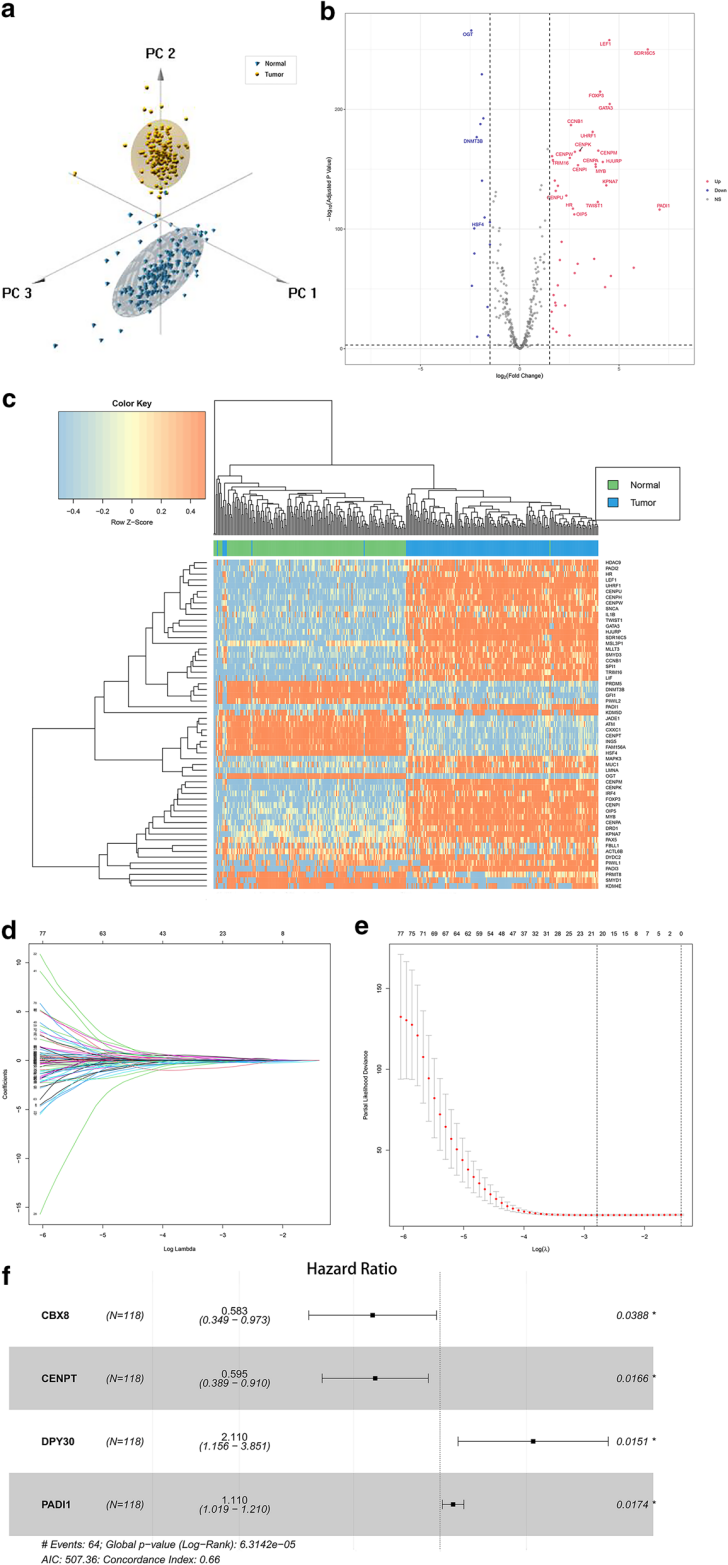
Screening out histone modification-related genes for constructing a risk signature. **a** PCA based on histone modification-related genes between normal pancreas and pancreatic cancer. **b** Volcano plot of histone modification-related DEGs in PC when compared with normal tissue. **c** Heatmap of histone modification-related DEGs between PC and normal tissue. **d** Log (Lambda) value of the 21 genes in LASSO regression analysis. **e** The most proper log (Lambda) value in LASSO regression analysis. **f** Four histone modification-related genes were screened out for constructing a risk signature

A flowchart of the analysis workflow was illustrated in Fig. 1. A total of 431 histone modification-related genes were integrated from the GSEA website (https://www.gsea-msigdb.org/gsea/index.jsp) (Additional file 3: Table S3). PCA analysis based on these histone modification-related genes confirmed the distribution difference between normal pancreas and pancreatic cancer in the TCGA + GTEx dataset. As shown in Fig. 2a, the clusters of tumor group were independent of normal group without obvious intersection (Fig. 2a). To further explore the specific histone modification-related differential genes between normal samples and tumor samples, we used edgeR packages to conduct differential analysis of them and made volcano plots and heatmaps. A total of 57 DEGs were identified, which included 42 upregulated genes and 15 downregulated genes (Fig. 2b, c). We then performed a univariate Cox regression analysis of 397 histone modification-related genes. Totally, 16 prognosis-related genes (HR > 1) and 24 prognosis-related genes (HR < 1) were found in the training set (n = 118). Subsequently, we combined the prognostic genes and DEGs to obtain a total of 82 candidate genes (Additional file 4: Table S4). LASSO regression analysis was performed on the candidate genes in order to avoid overfitting problems in risk signature, and 21 genes (*ACTL6A*, *CBX8*, *CENPA*, *CENPT*, *DPY30*, *DTX3L*, *DYDC2*, *FAM156A*, *FOXP3*, *HCFC1*, *HDAC4*, *ING5*, *JADE2*, *KDM8*, *LRWD1*, *MSL3P1*, *NAA50*, *PADI1*, *PAGR1*, *TWIST1*, *WDR5*) were retained according to the optimal lambda value (Fig. 2d, e, log(lambda.min) = − 2.787905). Random forest screening and multivariate Cox regression analysis were adopted to further identify an appropriate gene combination for establishing the risk signature for PC patients. Finally, 4 genes (*CBX8*, *CENPT*, *DPY30*, *PADI1*) were selected (Fig. 2f). Among these genes, CBX8 and CENPT were protective factors for PC survival with HR < 1, and DPY30 and PADI1 were risk factors with HR > 1.

### Construction of a risk signature for predicting 1-, 2- and 3-year survival rate of PC

**Fig. 3:**
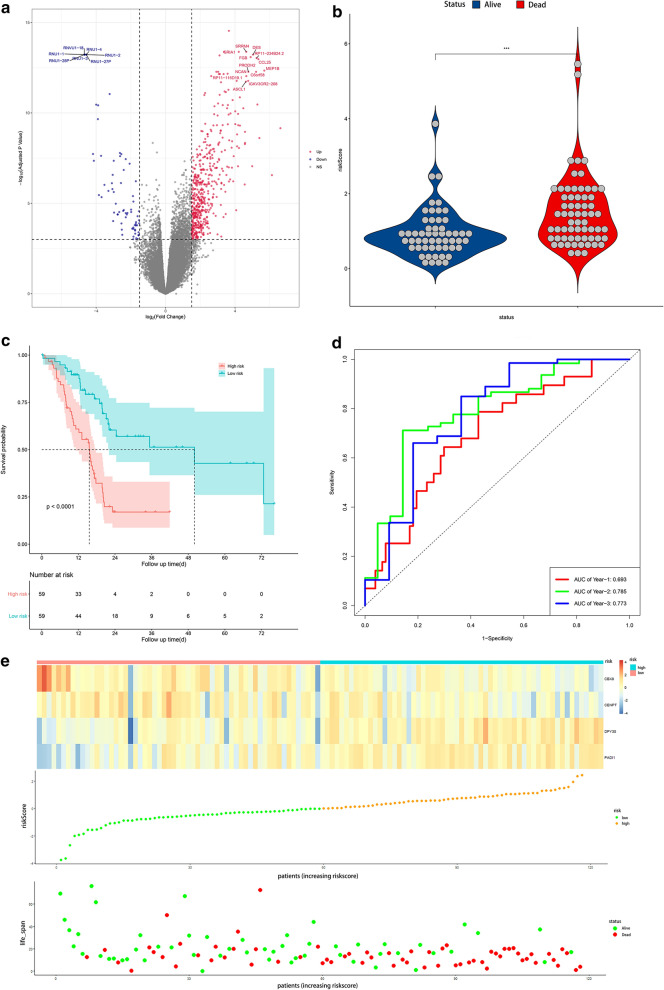
Construction of a risk signature in the training set. **a** Volcano plot of DEGs in high risk groups when compared with low risk groups. **b** Risk score comparison between the living and the dead, **c** Kaplan–Meier analysis of OS of the risk signature in training set. **d** Time-dependent ROC analysis of the risk signature in training set. **e** Heatmap of the four genes expression, the risk scores distribution and survival status of the patients in training set

Base on the expression level of four histone modification-related genes and the regression coefficient derived from the multivariate Cox regression model, we designed a risk-score formula for PC patients’ survival prediction in training set. The risk score for each patient was calculated as follows: Risk score = (0.1046 × expression level of PADI1) + (0.7465 × expression level of DPY30) + (− 0.5193 × expression level of CENPT) + (− 0.5399 × expression level of CBX8). According to the median cut-off value of the scores, the patients in the training set were divided into high-risk group (n = 59) and low-risk group (n = 59). Then DEGs analysis and the distribution of survival status illustrated the genomic distribution difference and prognostic differences between high and low risk groups (Fig. 3a, b). And genes with a P value < 1 × 10^–11^ and |logFC| > 4 were labelled in Fig. 3a. The Kaplan–Meier curves showed that patients in the high-risk group suffered worse prognosis than the patients in the low-risk group (Fig. 3c, P < 0.001).

To evaluate the competitive performance of the four histone modification-related genes signature, time-dependent ROC curve analysis was measured. As shown in the ROC curves, the area under curves (AUCs) of the risk signature were 0.693 for 1 year survival, 0.785 for 2 year survival and 0.773 for 3 year survival (Fig. 3d), proving a high prognostic value for survival prediction in the training dataset. Compared with the low-risk group, the expressions of CBX8 and CENPT in high-risk group decreased, while the expressions of DPY30 and PADI1 increased (Fig. 3e). At the same time, the number of deaths increased with the risk scores rising (Fig. 3e).

### Effectiveness and independence validation of the risk signature for the survival prediction

**Fig. 4:**
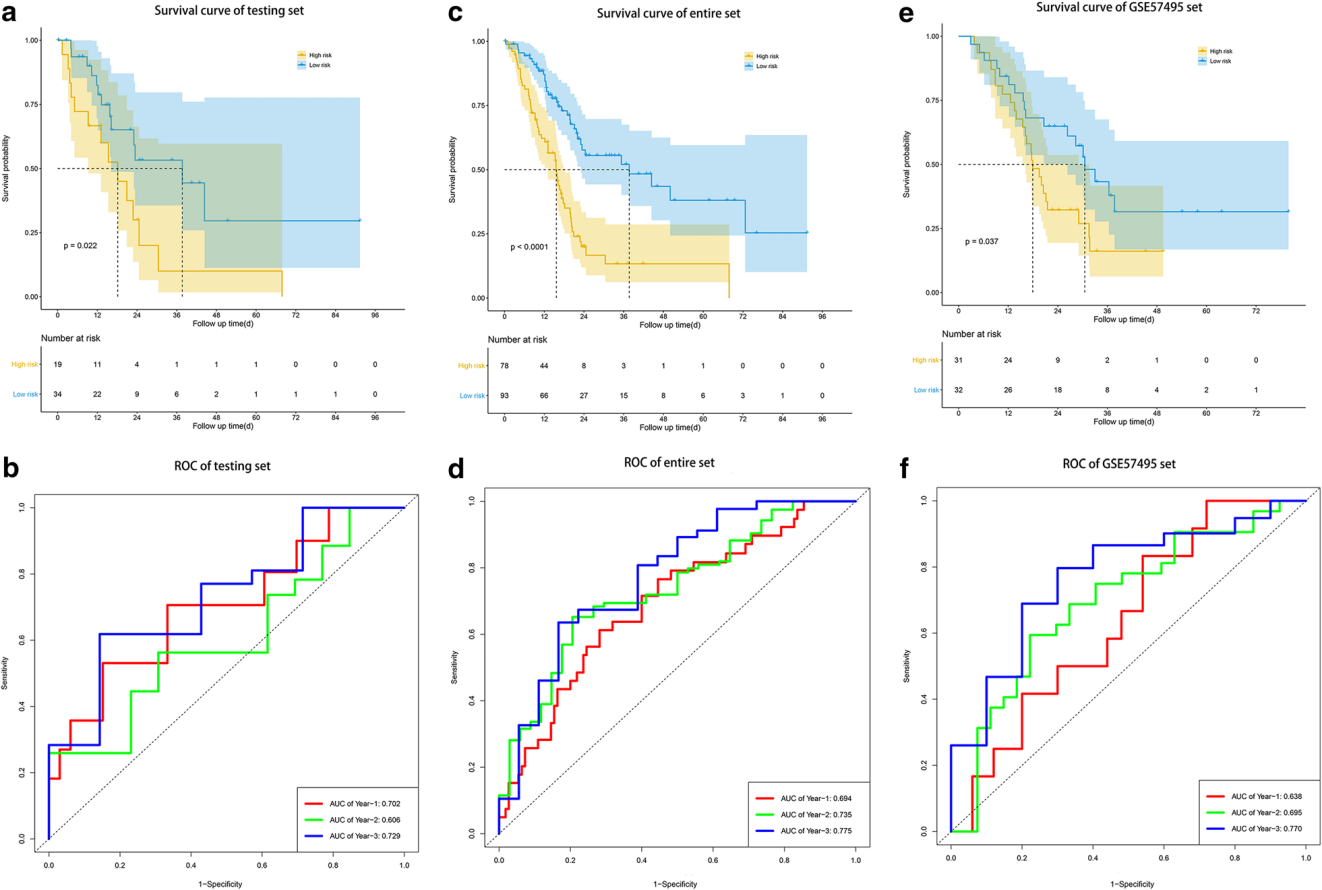
Validation of the risk signature for the survival prediction in testing set, the entire TCGA set and the GSE57495 set. **a**, **c**, **e** Kaplan–Meier analysis of OS of the risk signature in testing set, the entire TCGA set and the GSE57495 set. **b**, **d**, **f** Time-dependent ROC analysis of the risk signature in testing set, the entire TCGA set and the GSE57495 set

We next validated our risk signature in testing set (n = 53), the entire TCGA dataset (n = 171) and the GSE57495 dataset (n = 63) to confirm our findings. By calculating the risk scores for each patient based on the above-mentioned formula, the patients in these datasets were divided into high-risk group (n = 19 in testing set, n = 78 in entire set, n = 31 in GSE57495 set) and low risk group (n = 34 in testing set, n = 93 in entire set, n = 32 in GSE57495 set) using the same criteria. Consistent with the results in the training set, patients in the high-risk group had significantly poorer prognosis than those in the low risk group (Fig. 4a, testing set, P = 0.022; Fig. 4c, entire set, P < 0.001; Fig. 4e, GSE57495 set, P = 0.037). The AUCs of time dependent ROC curves for predicting 1-, 2- and 3-year survival of PC in the testing set were 0.702, 0.606 and 0.729, respectively (Fig. 4b), and those in the entire set were 0.694, 0.735, 0.775 (Fig. 4d). Meanwhile, the AUCs of time dependent ROC curves in the external validation set GSE57495 were 0.638, 0.695, 0.770 (Fig. 4e). Consistent with the results of the training set, the expressions of CBX8 and CENPT were decreased in the high-risk groups in three validation sets, while the expressions of DPY30 and PADI1 were increased. Concomitantly, as the risk score went up, the number of deaths increased (Additional file 5: Figure S1A, testing set; Additional file 5: Figure S1B, entire set; Additional file 5: Figure S1C, GSE57495 set), indicating that the risk signature performed well for predicting PC patients’ prognosis.

**Fig. 5:**
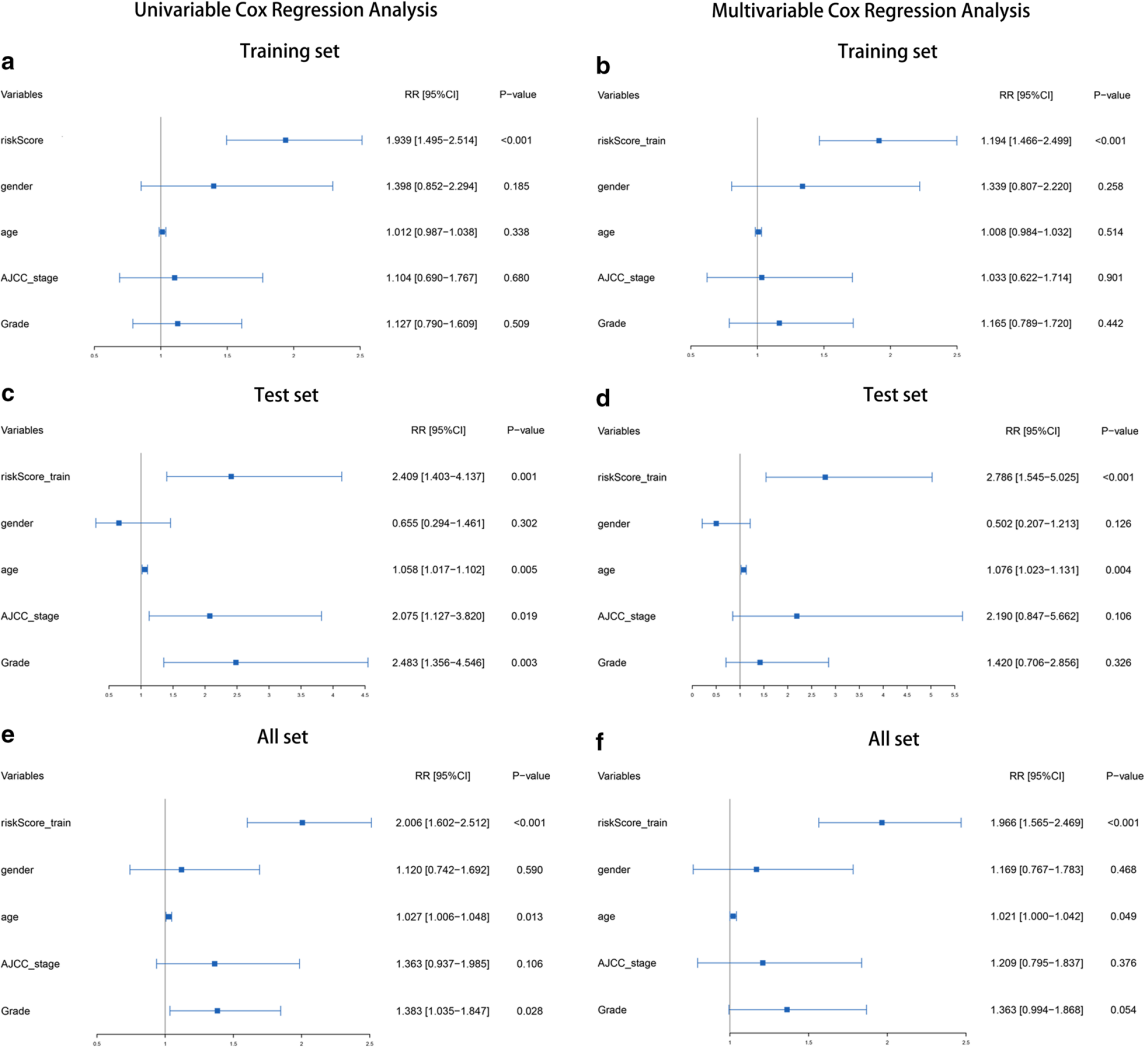
Independence of the risk signature and the other clinical variables, including gender, age, AJCC_stage and grade. **a**, **c**, **e** Univariate Cox regression analyses result in the training set, testing set and entire set. **b**, **d**, **f** Multivariate Cox regression analyses result in the training set testing set and entire set

Afterwards, we evaluated whether the survival prediction based on the risk signature was independent of clinical factors (Table 1). Univariate Cox regression analyses and multivariate Cox regression analyses were conducted on these factors in the training set, testing set and entire set respectively, which proved that the risk signature was an independent risk factor (Fig. 5a–f, P < 0.001 in all groups for risk score). We also explored the prognostic value of the risk signature in different cohorts stratified by age, gender, tumor grade and T stage (Additional file 6: Figure S2A–L). Regardless of the subgroup, patients in the high-risk group had significantly shorter survival times than those in the low-risk group, further demonstrating that the risk signature composed of four histone modification-related gene was an independent prognostic factor of PC.

In order to better optimize the risk signature, we also collected 171 PC patients in the TCGA dataset with detailed clinical information to construct and validate a nomogram. Finally, risk score, gender and N stages were incorporated into the construction of nomogram for predicting 1-, 2- and 3-year survival rate of PC (Additional file 7: Figure S3A–C). However, although the calibration plot and ROC curve both proved that the nomogram had a certain effect on predicting the prognosis of pancreatic cancer (Additional file 8: Figure S4A–L), its efficacy was not significantly better than that of the simple four gene risk signature, which may be related to the uniqueness of the risk signature itself and the bias of the sample.

Finally, we explored mutations in four key genes in the TCGA dataset (Additional file 9: Figure S5A). Moreover, in order to explain their mechanism of function, we explored their interaction network with the STRING database and verified their interaction in the TCGA dataset (Additional file 9: Figure S5B–E). Meanwhile, Pearson correlation analysis between risk scores and other genes in TCGA dataset was performed to reflect the comprehensive predictive ability of the four target genes (Additional file 10: Figure S6).

### Verification of the core gene of the risk signature in an independent cohort

**Fig. 6:**
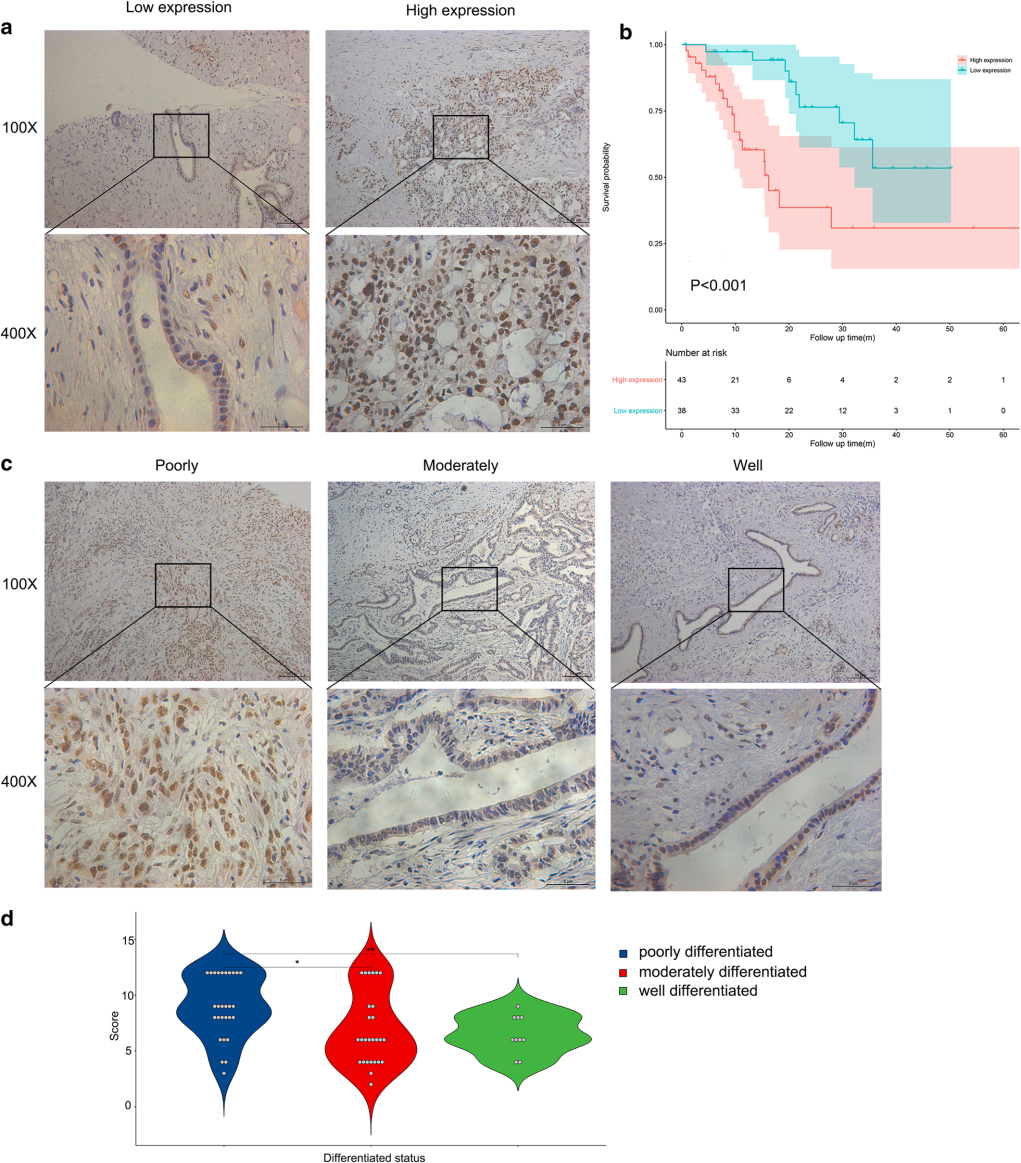
Verification of the core gene of the risk signature in an independent cohort. **a** Representative images of low and high expression of DPY30. **b** Kaplan–Meier analysis of OS of the expression of DPY30 in the independent cohort. **c** Representative images of poorly-, moderately-, and well-differentiated pancreatic cancer. **d** DPY30 expression levels in poorly-, moderately- and well-differentiated pancreatic cancers in the independent cohort

Among the four hub genes, DPY30 was accompanied by the smallest P value, the most significant HR, and the highest coefficient in the risk calculation formula, indicating that DPY30 occupied the most core position in the risk model. Therefore, we then validated the role of DPY30 expression in the prognosis of PC patients. We performed immunohistochemistry and calculated expression score of DPY30 in 81 PC patients. Combined with their clinical and pathologic data (Table 3), we further confirmed that patients with high DPY30 expression had lower overall survival (Fig. 6a, b, P < 0.001). Meanwhile, we also found that the expression of DPY30 in patients with moderately and highly differentiated pancreatic cancer was significantly lower than that in patients with poorly differentiated pancreatic cancer (Fig. 6c, d), further confirming that DPY30 accelerated the progression of pancreatic cancer.

**Table 3:** Clinical and pathologic information of the independent cohort

Character	Total (N = 81)		High expression (N = 43)		Low expression (N = 38)	
	Number	%	Number	%	Number	%
Age						
Median	63		64		63	
Range	47–83		48–83		47–78	
Score						
Median	8		9		6	
Range	1–12		8–12		1–6	
OS						
Median	15.5		9.8		20.4	
Range	0.7–68.9		0.8–68.9		0.7–50.2	
Gender						
Male	56	69.14	31	72.09	25	65.79
Female	25	30.86	12	27.91	13	34.21
Differentiation						
Well	10	12.35	4	9.30	6	15.79
Moderately	27	33.33	10	23.26	17	44.74
Poorly	28	34.57	22	51.16	6	15.79
Unknown	16	19.75	7	16.28	9	23.68

### Differential gene analysis, co-expression network construction and functional enrichments analysis between high and low risk groups

**Fig. 7:**
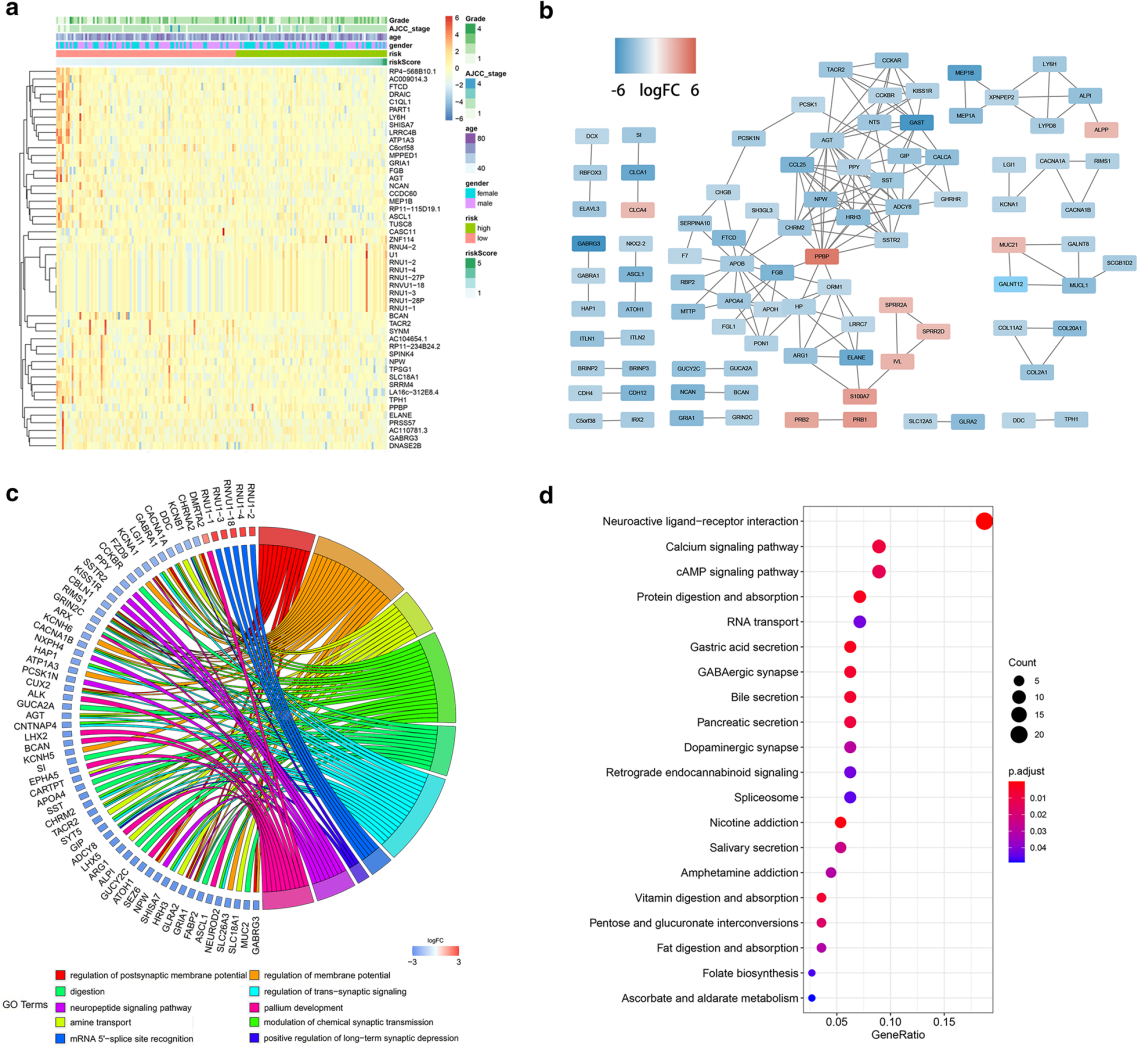
Differential gene analysis, co-expression network construction and functional enrichments analysis between high and low risk groups. **a** Heatmap of top 50 DEGs in PC between low and high-risk groups. **b** Co-expression network of DEGs constructed and visualized with STRING database and Cytoscape. **c** The risk signature-related top 10 GO enrichment based on DEGs between low and high risk groups. **d** The risk signature-related top 20 KEGG signaling pathway based on DEGs between low and high risk groups

Next, we explored the differences in gene expression between the high risk group (n = 78) and low risk groups (n = 93) in order to identify the mechanisms underlying the risk signature. DEGs were identified between high risk group and low risk group in TCGA entire set, |log2FC| > 2 and FDR < 0.001 were considered statistically significant. 50 genes with the most significant differences were shown in the heatmap and each patient’s clinical information was labeled at the top (Fig. 7a). Then the co-expression network was constructed and visualized with STRING database and Cytoscape (Fig. 7b). We set the minimum required interaction score to be high confidence (0.700) and hid disconnected nodes in the network, therefore not all genes were represented.

To further elucidate the molecular mechanism of the risk signature, we carried out GO and KEGG analyses of the above DEGs using clusterProfiler package in R (Fig. 7c, d). The top 10 pathways in the GO analysis and the top 20 pathways in the KEGG analysis were shown. At the same time, in order to reduce the bias, we performed GSEA analysis on all genes, whether or not they reached the difference threshold (Additional file 11: Figure S7A, B). Collectively, we noted that the main pathways enriched in GO analysis and KEGG analysis were the pathways associated with neuroendocrine interaction, hormonal regulation and metabolic function.

### CMap database analysis and drug sensitivity assay identify potential drugs targeting the risk signature

**Fig. 8:**
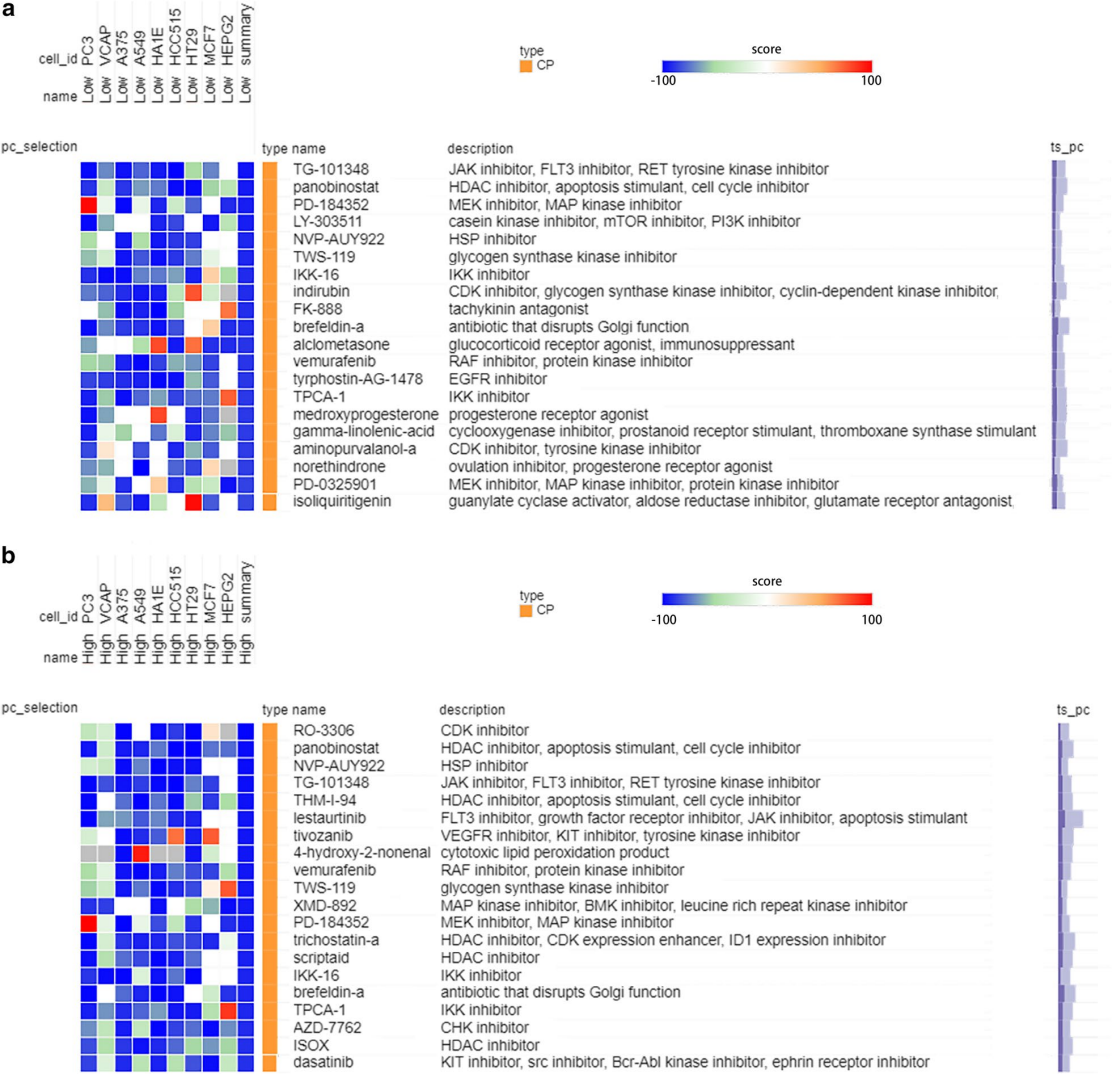
Small molecule drugs prediction by CMap database. **a** CMap database analysis identifies candidate drugs targeting the low-risk group, and the top 20 small molecule drugs with lower score are shown. **b** CMap database analysis identifies candidate drugs targeting the high-risk group, and the top 20 small molecule drugs with lower score are shown

Next, we utilized CMap database to compare the similarity of the DEGs profiles with the expression profiles of various small molecular compound treated cell lines aiming to identify novel candidate drugs as the risk model-related treatments for PC patients (Additional file 12: Figure S8). We used the heatmap to illustrate the similarity between the differential gene expression after drug treatment and the difference in the gene expression between the high and low risk groups (Fig. 8a, b). Figure 8a showed the potential small molecular drugs for the high-risk group patients, while Fig. 8b listed the potential drugs for the low-risk group patients. The top 20 small molecule drugs were shown in each figure.

**Fig. 9:**
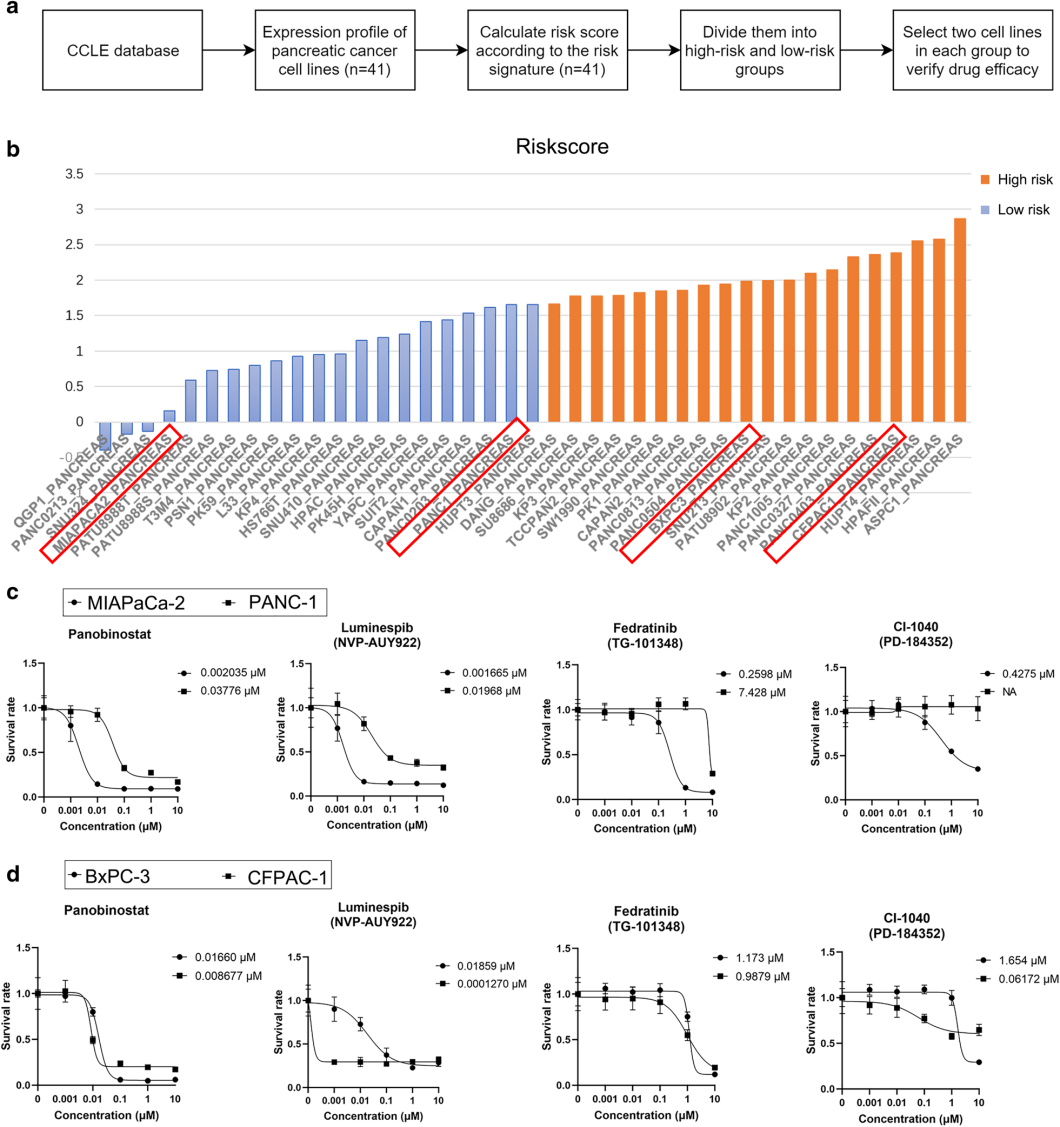
Validation of the efficacy of the drugs predicted by CMap database. **a** Flowchart of the drug validation process. **b** Risk score of pancreatic cancer cell lines and selected cell lines (red box). **c** Drug sensitivity curves and IC50 values of cell lines in the low-risk group. **d** Drug sensitivity curves and IC50 values of cell lines in the high-risk group

To further validate the efficacy of the drugs, drug sensitivity assay was performed on the drugs predicted by the CMap database (Fig. 9a). We noted that among the predicted drugs, panobinostat, luminespib (NVP-AUY922), fedratinib (TG-101348) and CI-1040 (PD-184352) were not only suitable for the treatment of PC patients in high-risk group but also in the low-risk group, implicating their potentiality in clinical usage. Therefore, we decided to verify their efficacy. All pancreatic cancer cell lines with expression data in Cancer Cell Line Encyclopedia (CCLE) database (https://portals.broadinstitute.org/ccle) were divided into high risk group and low risk group according to our risk model (Fig. 9b). Two cell lines were selected from each group to conduct drug sensitivity assay on the above four drugs. The results showed that panobinostat, luminespib and fedratinib exhibited excellent inhibitory effects on the growth of pancreatic cancer, especially panobinostat and luminespib, whose IC50 on the four cell lines was far less than that of other drugs (Fig. 9c, d). However, CI-1040 performed modest inhibitory effects PANC-1 cell lines, indicating that its therapeutic effect was relatively heterogeneous in patients (Fig. 9c, d).

## Discussion

PC is a highly lethal tumor due to the lack of early diagnosis and effective treatment. Currently, the radical surgical resection is still the most effective treatment. However, only less than 20% of all cases are resectable, and for those who undergo resection followed by adjuvant therapies, more than 80% will relapse and ultimately die of this disease [27]. There are many reasons for this dismal status, and one specific reason might be critical, the lack of an effective risk assessment models, which hinders the development of individualized treatment strategies for patients.

Extensive studies have recently revealed the key role of histone modification in the development of PC. The aberrant expression of histone modifiers may lead to a large-scale genetic remodeling in cells which accelerates pancreatic cancer occurrence and progression. Therefore, we explored the role of histone modification-related genes in predicting the prognosis of PC patients. In this study, we identified four histone modification related genes (CBX8, CENPT, DPY30, PADI1) associated with pancreatic cancer prognosis by a series of bioinformatics methods, including differential gene analysis, univariate and multivariate regression analysis, random forest screening, and LASSO regression analysis. Based on the expression of four histone modification-related genes and the regression coefficient derived from the multivariate Cox regression model, we designed a risk score formula for PC patients’ survival prediction. Furthermore, its ideal predictive efficiency was also verified in training set, testing set, entire set and external validation set. Furthermore, univariate Cox regression analyses and multivariate Cox regression analyses were conducted in the training set, testing set and entire set respectively, and proved that the risk signature was independent of clinical factors.

The four histone modification-related genes highlighted, including DPY30, CBX8, CENPT, and PADI1, have been reported to have the potential to regulate the occurrence and development of cancer to some extent by altering the histone modification of cancer-related genes.

DPY30, regarded as the core unit of the risk signature, is a common essential subunit of all Set1/MLL complexes and facilitates H3K4 methylation in cells [28, 29]. The WDR5, RbBP5, ASH2L, and DPY30 are collectively called WRAD, which make Set1/MLL complexes capable of di- and tri-methylation activities [30], while MLL by itself is a mono-methyltransferase [31]. Multiple studies have shown that DPY30 plays a pivotal role under physiological condition. Jiang et al. has demonstrated that DPY30 directly regulates chromosomal H3K4 trimethylation throughout the mammalian genome, which significantly alters the differentiation potential of embryonic stem cells (ESCs), particularly along the neural lineage [32]. And two other studies also revealed that DPY30 played a critical role in the proliferation, differentiation and function of hematopoietic stem cells by affecting the H3K4 methylation activity of the Set1/MLL complex [33, 34]. Therefore, it is reasonable to speculate that the abnormal regulation of DPY30 will lead to aberrant methylation of histones, which may cause the disturbance of body homeostasis and eventually lead to the occurrence of cancer. A study of Burkitt lymphoma further confirmed our hypothesis. Researchers demonstrated that DPY30 promoted the expression of endogenous MYC and was functionally important for efficient binding of MYC to its genomic targets by regulating chromatin accessibility [35]. In our risk signature, we also screened DPY30 as one of the risk factors for predicting the prognosis of PC and verified it in our cohort, further supporting previous researches.

CBX8, one of the core components of canonical polycomb repressive complex 1 (PRC1) [36], has been reported in recent years to be involved in the process of histone modification, especially histone methylation, so as to regulate the occurrence and development of tumors. For example, EZH2, a catalytic subunit of polycomb repressive complex 2 (PRC2) and one of the most reported methyltransferases which represses gene expression via methylation of lysine 27 of histone 3 (H3K27) [37, 38], requires the presence of CBX8 for its biological actions in driving germinal center formation and lymphoma precursor lesions [39]. In addition, CBX8 recruits KMT2B (lysine methyltransferases 2B) to the LGR5 promoter, which maintains H3K4me3 status to promote LGR5 expression, resulting in increased cancer stemness and decreased chemosensitivity in colon cancer [40]. However, the specific mechanism by which CBX8 interacts with histone methylation is also elusive. On the one hand, the CBX8 chromodomains demonstrates the weakest histone peptide binding of the five CBX chromodomains and no measurable specificity for H3K27me3 peptides [41]. On the other hand, however, studies also revealed that H3K27me3 was crucial for the association of CBX8 and chromatin [42–44]. In addition, the role of CBX8 in tumors is also paradoxical, which means CBX8 may also exhibit antitumor effects under certain conditions. In the metastasis of esophageal squamous cell carcinoma, CBX8 serves as a tumor suppressor by binding with the Snail promoter and suppressing the transcription of Snail [45]. And our results also support its protective effect for human body. Meanwhile, although the role of CBX8 in several cancers is explained such as hepatocellular carcinoma and acute myeloid leukemia [46, 47], the function and association with histone modification have not been reported in pancreatic cancer yet, which needs a further exploration.

Compared with CBX8 and DPY30, CENPT and PDAI1 have been less reported on promoting tumor development by influencing histone modification. CENPT is a histone fold-containing protein, which is able to form a heterotetrameric nucleosome-like structure together with three other histone fold-containing proteins, CENPW, CENPS and CENPX. These tetrameric nucleosome-like structures extend the “histone code” beyond the canonical nucleosome proteins to provide a new mechanism to form contacts with DNA [48]. Meanwhile, CENPT is also required for the kinetochore assembly function of CENPA [49–51], a CenH3 (centromere-specific histone H3) variant which replace histone H3 to form specialized chromatin that acts as the foundation for kinetochore assembly [52]. Another study also identified the CENPT/CENPW complex as a binding partner of the histone chaperone FACT [53]. Therefore, CENPT plays an important role in the integrity of the genetic material of cells during mitosis, and its aberrant expression may lead to tumor progression. In our predictive signature, CENPT acts as a protective factor, indicating that it plays a role in protecting the integrity of genetic material in the development of PC. Moreover, another study conducted by Giunta et al. further confirmed our view [54].

Finally, the PADI1 gene is a member of the peptidylarginine deiminases (PADIs) family, which contains a cluster of calcium dependent enzymes that catalyzes the conversion of arginine residues to citrulline residues via a hydrolytic process termed citrullination in proteins including histone [55, 56]. However, while a growing number of researches have shown that PADI-mediated histone citrullination is highly associated with cancer development [57–59], PADI1 has been the one that is overlooked. Although the role of PADI1 in cancer progression has also been elucidated [60], and PADI1-mediated histone citrullination is crucial in early embryonic development [61], it remains to be explored whether PADI1-mediated histone citrullination play a role in cancer development.

Histone modification-related proteins functions mainly through the histone modification of tumor-related genes in cancer occurrence and development. Therefore, their relationship with each other is actually the cross-linking interaction between various histone modifications. Among the four target genes, CBX8 is involved in the ubiquitination of H2AK119 as part of polycomb repressive complex 1 (PRC1) [36], DPY30 is involved in the methylation of H3K4 as part of SET1/MLL complex [28, 29], and PADI1 is involved in the citrullination of H4R3 and H3R2/8/17 [61]. They are one of the factors that maintain the homeostasis of histone modifications in the body, or near certain tumor-related genes. When one of these changes, it will directly or indirectly lead to abnormal changes in the other several modifications. For instance, Beta-Hydroxybutyrate enhances brain-derived neurotrophic factor (BDNF) expression by increasing H3K4me3 and decreasing H2AK119ub occupancy at the BDNF promoters in hippocampal neurons [62]. In addition, the ubiquitination of H2AK119 in cancer cells recruits PRC2 for H3K27 methylation, thereby antagonizing the H3K4me3-mediated MHC class I (MHC-I) activation [63, 64]. Moreover, CENPT, as one of the four target gene members, seems have little correlation with the other three genes according to the researches so far. CENPT plays a central role in assembly of kinetochore proteins, mitotic progression and chromosome segregation [49–51], thus ensuring the stability and integrity of the genetic material of cells during mitosis. These findings suggest that CBX8, DPY30, and PADI1 may be used as a cluster to predict pancreatic cancer prognosis. And the relationship between CENPT and them is still unclear, either as an individual predictor or because current studies have not fully revealed its role.

To further demonstrate whether these four target genes and the risk signature they constitute are indeed prognostic factors, we further explored their association with other genes, especially cancer-related genes. Regarding the four target genes, we explored their interaction network with the STRING database and verified their correlation according to Pearson correlation analysis in our TCGA dataset. We found that two risk genes, DPY30 and PADI1, were significantly positively associated with several identified cancer-related genes. For example, DPY30 was significantly positively correlated with SETD1A, SETDB1 and RBBP5, which have been reported to promote the occurrence and progression of cancers [65–67]. Meanwhile, PADI1 also showed a similar trend, and its expression was also positively correlated with a series of tumor promoting genes, such as STAP2 and DOCK1 [68, 69], etc. As for the other two protective genes, we also found that they were positively associated with a number of tumor suppressor genes. A significant positive correlation between CENPT and ITGB3BP was observed, while a positive correlation between CBX8 and PCGF2 was found. And ITGB3BP and PCGF2 have been reported to inhibit the occurrence, proliferation, metastasis and drug resistance of tumors [70–72].

In order to reflect the comprehensive predictive ability of the four target genes, we also performed Pearson correlation analysis between risk scores and other genes in TCGA dataset (see Additional file 9: Figure S5). A number of widely reported cancer related genes have been revealed, including DPY30, EREG, PLA2G16, ZNF185, PADI1, CDK3, INPP4B, DPH1, AP1S3 and so on. Among them, DPY30, EREG, PLA2G16, ZNF185, PADI1, INPP4B and AP1S3 have been reported to be involved in tumor genesis and progression [60, 73–78], and they were significantly positively correlated with risk score. In addition, CDK3 and DPH1, which have been reported to inhibit or have the potential to inhibit tumor development [79, 80], was observed to be markedly negatively correlated with risk scores.

Subsequently, differential gene analysis, PPI network mapping and functional enrichment analysis were performed for patients in the high and low risk group. Collectively, we noted that the main pathways enriched in GO analysis and KEGG analysis were the pathways associated with neuroendocrine interaction, hormonal regulation and metabolic function. Moreover, in order to reduce the bias, we performed GSEA analysis on all genes, whether or not they reached the difference threshold. And the result of GSEA analysis was also consistent with the results above. Therefore, we speculated that the mechanism of poor prognosis in high-risk patients may be related to the insufficient insulin secretion caused by neuroendocrine abnormality, which mediates metabolic disorders in vivo. We can assume that in high-risk group, neuroendocrine abnormalities lead to impaired endocrine function in the pancreas, particularly the production of insulin. Mechanically, the neuroactive ligand-receptor interaction, the calcium signaling pathway and cAMP signaling pathway are significantly down-regulated, which have been proved to be the main mechanisms of insulin secretion [81–83]. Insufficient insulin secretion will lead to a series of metabolic disorders in vivo such as hyperglycemia, and further aggravate the progression of pancreatic cancer [84, 85]. This effect is also exacerbated by the fact that most pancreatic cancer patients suffer cancer-related or -unrelated diabetes [86]. Although most pancreatic cancer patients are treated with glucose-lowering drugs or insulin, it is still different from their natural physical condition, and insulin is also an essential factor in promoting the development of PC [87, 88]. Taken together, we hypothesized that the primary cause of poor prognosis of pancreatic cancer, at least in our study, was metabolic disorders caused by inadequate insulin secretion, which was fueled by neuroendocrine aberration. At the same time, a list of studies has reported that the prognosis of patients with pancreatic cancer complicated with diabetes is significantly worse than that of patients without diabetes [89, 90], further confirming our opinion.

More importantly, utilizing CMap database, we identified a range of small molecule compounds, which might ultimately pave the way for implementation of targeting the risk model-related treatments for PC patients. Among them, the most common compounds are HDAC inhibitors, including panobinostat, scriptaid, trichostatin-a, ISOX and THM-I-94. Notably, the potential of HDAC inhibitors in the treatment of pancreatic cancer has received increasing attention in recent years [91–93], and relevant clinical trials have been carried out [94]. These results further highlight the critical role of histone modification in the prognosis of PC.

Meanwhile, through drug sensitivity experiments, we further confirmed the inhibitory effect of panobinostat, luminespib, fedratinib and CI-1040 on pancreatic cancer growth. Although some effects have been reported [95, 96], we selected cell lines with different risk levels for verification according to our constructed risk signature, which further confirmed the wide coverage of drug effectiveness and laid a solid foundation for further in-depth mechanism studies and clinical trials in the future.

Nevertheless, several limitations in our study should be acknowledged. First, because of the extremely poor prognosis of PC, the survival time of the sample is rarely longer than 3 years, which may lead to the inaccurate results when we want to predict the long-term outcome of the patients. Second, due to the limited clinical information in the GSE57495 dataset, we are unable to verify the independence of the risk model and validate the nomogram in this dataset. Third, although we tried our best to avoid it through Short Tandem Repeat (STR) identification and mycoplasma detection, the use of the algorithm based on clinical samples on immortalized cell lines may cause some deviation due to the fact that the immortalized cell lines have undergone a certain amount of passage and may have altered genetic signatures.

## Conclusions

In summary, a risk signature consisting of four histone modification-related genes was constructed in our study, including CBX8, CENPT, DPY30 and PADI1. We further validated its predictive performance and proved it as an independent prognostic factor. Mechanically, functional enrichment analysis showed that the risk signature prediction might be relevant to the metabolic disorders caused by insufficient insulin secretion, which was fueled by neuroendocrine abnormality. Finally, a cluster of small molecule drugs were identified as the risk model-related treatments for PC patients by CMap database and drug sensitivity assay. Our findings will help to accurately assess the prognosis of PC patients and optimize the clinical decision-making.

## Supplementary Information

**Additional file 1: Table S1.** Clinical information of samples in TCGA entire dataset.

**Additional file 2: Table S2.** Clinical information of samples in GSE57495 dataset.

**Additional file 3: Table S3.** Genes related to histone modification obtained from the GSEA website.

**Additional file 4: Table S4.** Candidate genes obtained by differential gene expression analysis and univariate regression analysis.

**Additional file 5: Figure S1.** Further validation of the risk signature for the survival prediction in testing set, the entire TCGA set and the GSE57495 set. (A–C) Heatmap of the four genes expression, the risk scores distribution and survival status of the patients in the testing set, entire TCGA set and GSE57495 set.

**Additional file 6: Figure S2.** Stratification analyses of all patients using the risk signature. (A–C) The Kaplan–Meier plot of the younger stratum (age ≤ 65, n = 91), older stratum (age > 65, n = 80) and entire patients with PC (n = 171). (D–F) The Kaplan–Meier plot of the male stratum (n = 93), female stratum n = 78) and entire patients with PC (n = 171). (G–I) The Kaplan–Meier plot of the Grade I/II stratum (n = 121), Grade III/IV stratum (n = 50) and entire patients with PC (n = 171). (J–L) The Kaplan–Meier plot of the T1 + T2 stratum (n = 30), T3 + T4 stratum (n = 141) and entire patients with PC (n = 171).

**Additional file 7: Figure S3.** Construction of a nomogram for predicting 1-, 2- and 3-year survival rate of PC. (A) Forrest plot of univariate Cox regression analysis in training set. (B) Forrest plot of multivariate Cox regression analysis in in training set. (C) Nomogram integrating four histone modification gene-based risk score, gender and N stage.

**Additional file 8: Figure S4.** Validation of the nomogram in training set, testing set and entire set. (A–C) Time-dependent ROC analysis of the risk signature in training set, testing set and entire set. (D–L) The calibration plot of the nomogram for agreement test between 1-, 2- and 3-year OS prediction and actual outcome in the training set, testing set and entire set.

**Additional file 9: Figure S5.** The mutations status and mechanisms of function of DPY30, CBX8, CENPT and PADI1. (A) Mutation information of DPY30, CBX8, CENPT and PADI1 from the cBioPortal database. (B–E) Interaction of DPY30, CBX8, CENPT and PADI1 with other proteins obtained from STRING database and validation in the TCGA entire set.

**Additional file 10: Figure S6.** Correlation between risk score and expression of tumor-related genes through Pearson correlation analysis in TCGA dataset.

**Additional file 11: Figure S7.** Gene Set Enrichment Analysis (GSEA) based on all genes between low and high risk groups. The top 3 GO enrichments (A) and KEGG enrichments (B) are displayed on the top respectively.

**Additional file 12: Figure S8.** Differential gene expression in the high- and low-risk group compared with the normal group. (A) Heatmap of DEGs (Top50) between the low-risk pancreatic cancer group and the normal group. (B) Volcanic map of the DEGs between the low-risk pancreatic cancer group and the normal group. (C) Heatmap of DEGs (Top50) between the high-risk pancreatic cancer group and the normal group. (D) Volcanic map of the DEGs between the high-risk pancreatic cancer group and the normal group

## Data Availability

Not applicable.

## Abbreviations

PC: Pancreatic cancer
CBX8: Chromobox 8
CENPT: Centromere Protein T
DPY30: Dpy-30 Histone Methyltransferase Complex Regulatory Subunit
PADI1: Peptidyl Arginine Deiminase 1
TCGA: The Cancer Genome Atlas
GTEx: Genotype-Tissue Expression
GEO: Gene Expression Omnibus
DEGs: Differential expressed genes
LASSO: Least absolute shrinkage and selection operator
ROC curve: Receiver operating characteristic curve
KM: Kaplan–Meier
AUCs: Areas under curve
AJCC: American Joint Committee on Cancer
lncRNA: Long non-coding RNA
ceRNA: Competing endogenous RNAs
GSEA: Gene Set Enrichment Analysis
TPM: Transcripts Per Kilobase Million
RMA: Robust multi-array average
PCA: Principal component analysis
FDR: False discovery rate
FC: Fold change
OS: Overall survival
EDTA: Ethylene Diamine Tetraacetic Acid
GO: Gene Ontology
KEGG: Kyoto Encyclopedia of Genes and Genomes
ATCC: American Type Culture Collection
DMSO: Dimethyl sulfoxide
CCK-8: Cell Counting Kit-8
HR: Hazard ratio
CCLE: Cancer Cell Line Encyclopedia
H3K27me3: Trimethylation of histone 3 lysine 27
H3K9me2: Dimethylation of histone 3 lysine 9
H3K4me3: Trimethylation of histone 3 lysine 4
H3K27ac: Monoacetylation of histone 3 lysine 27
ESCs: Embryonic stem cells
PRC1/2: Polycomb repressive complex 1/2
EZH2: Enhancer of zeste homolog 2
KMT2B: Lysine methyltransferases 2B
CenH3: Centromere-specific histone H3
HDAC: Histone deacetylase

## References

[1] Siegel RL, Miller KD, Fuchs HE, Jemal A (2021). Cancer statistics, 2021. CA Cancer J Clin.

[2] Sung H, Ferlay J, Siegel RL, Laversanne M, Soerjomataram I, Jemal A, Bray F (2021). Global cancer statistics 2020: GLOBOCAN estimates of incidence and mortality worldwide for 36 cancers in 185 countries. CA Cancer J Clin.

[3] Mizrahi JD, Surana R, Valle JW, Shroff RT (2020). Pancreatic cancer. Lancet.

[4] Tempero MA (2019). NCCN guidelines updates: pancreatic cancer. JNCCN.

[5] van Roessel S, Kasumova GG, Verheij J, Najarian RM, Maggino L, de Pastena M, Malleo G, Marchegiani G, Salvia R, Ng SC (2018). International validation of the eighth edition of the American Joint Committee on Cancer (AJCC) TNM staging system in patients with resected pancreatic cancer. JAMA Surg.

[6] Allen PJ, Kuk D, Castillo CF, Basturk O, Wolfgang CL, Cameron JL, Lillemoe KD, Ferrone CR, Morales-Oyarvide V, He J (2017). Multi-institutional validation study of the American Joint Commission on Cancer (8th edition) changes for T and N staging in patients with pancreatic adenocarcinoma. Ann Surg.

[7] Marquardt JU, Galle PR, Teufel A (2012). Molecular diagnosis and therapy of hepatocellular carcinoma (HCC): an emerging field for advanced technologies. J Hepatol.

[8] Koboldt DC, Steinberg KM, Larson DE, Wilson RK, Mardis ER (2013). The next-generation sequencing revolution and its impact on genomics. Cell.

[9] Reyna MA, Haan D, Paczkowska M, Verbeke LPC, Vazquez M, Kahraman A, Pulido-Tamayo S, Barenboim J, Wadi L, Dhingra P (2020). Pathway and network analysis of more than 2500 whole cancer genomes. Nat Commun.

[10] Barroso-Sousa R, Keenan TE, Pernas S, Exman P, Jain E, Garrido-Castro AC, Hughes M, Bychkovsky B, Umeton R, Files JL (2020). Tumor mutational burden and PTEN alterations as molecular correlates of response to PD-1/L1 blockade in metastatic triple-negative breast cancer. Clin Cancer Res.

[11] Zhou Z, Cheng Y, Jiang Y, Liu S, Zhang M, Liu J, Zhao Q (2018). Ten hub genes associated with progression and prognosis of pancreatic carcinoma identified by co-expression analysis. Int J Biol Sci.

[12] Zhou YY, Chen LP, Zhang Y, Hu SK, Dong ZJ, Wu M, Chen QX, Zhuang ZZ, Du XJ (2019). Integrated transcriptomic analysis reveals hub genes involved in diagnosis and prognosis of pancreatic cancer. Mol Med.

[13] Wu M, Li X, Zhang T, Liu Z, Zhao Y (2019). Identification of a nine-gene signature and establishment of a prognostic nomogram predicting overall survival of pancreatic cancer. Front Oncol.

[14] Song J, Xu Q, Zhang H, Yin X, Zhu C, Zhao K, Zhu J (2018). Five key lncRNAs considered as prognostic targets for predicting pancreatic ductal adenocarcinoma. J Cell Biochem.

[15] Kandimalla R, Tomihara H, Banwait JK, Yamamura K, Singh G, Baba H, Goel A (2020). A 15-gene immune, stromal, and proliferation gene signature that significantly associates with poor survival in patients with pancreatic ductal adenocarcinoma. Clin Cancer Res.

[16] Wang W, Lou W, Ding B, Yang B, Lu H, Kong Q, Fan W (2019). A novel mRNA-miRNA-lncRNA competing endogenous RNA triple sub-network associated with prognosis of pancreatic cancer. Aging.

[17] Feinberg AP (2018). The key role of epigenetics in human disease prevention and mitigation. N Engl J Med.

[18] Roe JS, Hwang CI, Somerville TDD, Milazzo JP, Lee EJ, Da Silva B, Maiorino L, Tiriac H, Young CM, Miyabayashi K (2017). Enhancer reprogramming promotes pancreatic cancer metastasis. Cell.

[19] Comet I, Riising EM, Leblanc B, Helin K (2016). Maintaining cell identity: PRC2-mediated regulation of transcription and cancer. Nat Rev Cancer.

[20] Nacev BA, Feng L, Bagert JD, Lemiesz AE, Gao J, Soshnev AA, Kundra R, Schultz N, Muir TW, Allis CD (2019). The expanding landscape of 'oncohistone' mutations in human cancers. Nature.

[21] Leek JT, Storey JD (2007). Capturing heterogeneity in gene expression studies by surrogate variable analysis. PLoS Genet.

[22] Robinson MD, McCarthy DJ, Smyth GK (2010). edgeR: a Bioconductor package for differential expression analysis of digital gene expression data. Bioinformatics.

[23] Harris MA, Clark J, Ireland A, Lomax J, Ashburner M, Foulger R, Eilbeck K, Lewis S, Marshall B, Mungall C (2004). The Gene ontology (GO) database and informatics resource. Nucleic Acids Res.

[24] Kanehisa M, Goto S (2000). KEGG: kyoto encyclopedia of genes and genomes. Nucleic Acids Res.

[25] Subramanian A, Tamayo P, Mootha VK, Mukherjee S, Ebert BL, Gillette MA, Paulovich A, Pomeroy SL, Golub TR, Lander ES (2005). Gene set enrichment analysis: a knowledge-based approach for interpreting genome-wide expression profiles. Proc Natl Acad Sci USA.

[26] Yu G, Wang LG, Han Y, He QY (2012). clusterProfiler: an R package for comparing biological themes among gene clusters. OMICS.

[27] Kleeff J, Korc M, Apte M, La Vecchia C, Johnson CD, Biankin AV, Neale RE, Tempero M, Tuveson DA, Hruban RH (2016). Pancreatic cancer. Nat Rev Dis Prim.

[28] Dou Y, Milne TA, Ruthenburg AJ, Lee S, Lee JW, Verdine GL, Allis CD, Roeder RG (2006). Regulation of MLL1 H3K4 methyltransferase activity by its core components. Nat Struct Mol Biol.

[29] Kwon M, Park K, Hyun K, Lee JH, Zhou L, Cho YW, Ge K, Skalnik DG, Muir TW, Kim J (2020). H2B ubiquitylation enhances H3K4 methylation activities of human KMT2 family complexes. Nucleic Acids Res.

[30] Zhang Y, Mittal A, Reid J, Reich S, Gamblin SJ, Wilson JR (2015). Evolving catalytic properties of the MLL family SET domain. Structure.

[31] Patel A, Vought VE, Dharmarajan V, Cosgrove MS (2008). A conserved arginine-containing motif crucial for the assembly and enzymatic activity of the mixed lineage leukemia protein-1 core complex. J Biol Chem.

[32] Jiang H, Shukla A, Wang X, Chen WY, Bernstein BE, Roeder RG (2011). Role for Dpy-30 in ES cell-fate specification by regulation of H3K4 methylation within bivalent domains. Cell.

[33] Yang Z, Augustin J, Chang C, Hu J, Shah K, Chang CW, Townes T, Jiang H (2014). The DPY30 subunit in SET1/MLL complexes regulates the proliferation and differentiation of hematopoietic progenitor cells. Blood.

[34] Yang Z, Shah K, Khodadadi-Jamayran A, Jiang H (2016). Dpy30 is critical for maintaining the identity and function of adult hematopoietic stem cells. J Exp Med.

[35] Yang Z, Shah K, Busby T, Giles K, Khodadadi-Jamayran A, Li W, Jiang H (2018). Hijacking a key chromatin modulator creates epigenetic vulnerability for MYC-driven cancer. J Clin Investig.

[36] Laugesen A, Helin K (2014). Chromatin repressive complexes in stem cells, development, and cancer. Cell Stem Cell.

[37] Cao R, Wang L, Wang H, Xia L, Erdjument-Bromage H, Tempst P, Jones RS, Zhang Y (2002). Role of histone H3 lysine 27 methylation in Polycomb-group silencing. Science.

[38] McCabe MT, Ott HM, Ganji G, Korenchuk S, Thompson C, Van Aller GS, Liu Y, Graves AP, Della Pietra A, Diaz E (2012). EZH2 inhibition as a therapeutic strategy for lymphoma with EZH2-activating mutations. Nature.

[39] Béguelin W, Teater M, Gearhart MD, Calvo Fernández MT, Goldstein RL, Cárdenas MG, Hatzi K, Rosen M, Shen H, Corcoran CM (2016). EZH2 and BCL6 cooperate to assemble CBX8-BCOR complex to repress bivalent promoters, mediate germinal center formation and lymphomagenesis. Cancer Cell.

[40] Zhang Y, Kang M, Zhang B, Meng F, Song J, Kaneko H, Shimamoto F, Tang B (2019). m(6)A modification-mediated CBX8 induction regulates stemness and chemosensitivity of colon cancer via upregulation of LGR5. Mol Cancer.

[41] Kaustov L, Ouyang H, Amaya M, Lemak A, Nady N, Duan S, Wasney GA, Li Z, Vedadi M, Schapira M (2011). Recognition and specificity determinants of the human cbx chromodomains. J Biol Chem.

[42] Connelly KE, Weaver TM, Alpsoy A, Gu BX, Musselman CA, Dykhuizen EC (2019). Engagement of DNA and H3K27me3 by the CBX8 chromodomain drives chromatin association. Nucleic Acids Res.

[43] Zhen CY, Tatavosian R, Huynh TN, Duc HN, Das R, Kokotovic M, Grimm JB, Lavis LD, Lee J, Mejia FJ (2016). Live-cell single-molecule tracking reveals co-recognition of H3K27me3 and DNA targets polycomb Cbx7-PRC1 to chromatin. Elife.

[44] Wang S, Denton KE, Hobbs KF, Weaver T, McFarlane JMB, Connelly KE, Gignac MC, Milosevich N, Hof F, Paci I (2020). Optimization of ligands using focused DNA-encoded libraries to develop a selective, cell-permeable CBX8 chromodomain inhibitor. ACS Chem Biol.

[45] Wang G, Tang J, Zhan W, Zhang R, Zhang M, Liao D, Wang X, Wu Y, Kang T (2017). CBX8 suppresses tumor metastasis via repressing snail in esophageal squamous cell carcinoma. Theranostics.

[46] Zhang CZ, Chen SL, Wang CH, He YF, Yang X, Xie D, Yun JP (2018). CBX8 exhibits oncogenic activity via AKT/β-catenin activation in hepatocellular carcinoma. Can Res.

[47] Tan J, Jones M, Koseki H, Nakayama M, Muntean AG, Maillard I, Hess JL (2011). CBX8, a polycomb group protein, is essential for MLL-AF9-induced leukemogenesis. Cancer Cell.

[48] Nishino T, Takeuchi K, Gascoigne KE, Suzuki A, Hori T, Oyama T, Morikawa K, Cheeseman IM, Fukagawa T (2012). CENP-T-W-S-X forms a unique centromeric chromatin structure with a histone-like fold. Cell.

[49] Ding M, Jiang J, Yang F, Zheng F, Fang J, Wang Q, Wang J, Yao W, Liu X, Gao X (2019). Holliday junction recognition protein interacts with and specifies the centromeric assembly of CENP-T. J Biol Chem.

[50] Thakur J, Henikoff S (2016). CENPT bridges adjacent CENPA nucleosomes on young human α-satellite dimers. Genome Res.

[51] Hara M, Ariyoshi M, Okumura EI, Hori T, Fukagawa T (2018). Multiple phosphorylations control recruitment of the KMN network onto kinetochores. Nat Cell Biol.

[52] Foltz DR, Jansen LE, Black BE, Bailey AO, Yates JR, Cleveland DW (2006). The human CENP-A centromeric nucleosome-associated complex. Nat Cell Biol.

[53] Prendergast L, Müller S, Liu Y, Huang H, Dingli F, Loew D, Vassias I, Patel DJ, Sullivan KF, Almouzni G (2016). The CENP-T/-W complex is a binding partner of the histone chaperone FACT. Genes Dev.

[54] Giunta S, Funabiki H (2017). Integrity of the human centromere DNA repeats is protected by CENP-A, CENP-C, and CENP-T. Proc Natl Acad Sci USA.

[55] Arita K, Shimizu T, Hashimoto H, Hidaka Y, Yamada M, Sato M (2006). Structural basis for histone N-terminal recognition by human peptidylarginine deiminase 4. Proc Natl Acad Sci USA.

[56] Klose RJ, Zhang Y (2007). Regulation of histone methylation by demethylimination and demethylation. Nat Rev Mol Cell Biol.

[57] Zhang X, Bolt M, Guertin MJ, Chen W, Zhang S, Cherrington BD, Slade DJ, Dreyton CJ, Subramanian V, Bicker KL (2012). Peptidylarginine deiminase 2-catalyzed histone H3 arginine 26 citrullination facilitates estrogen receptor α target gene activation. Proc Natl Acad Sci USA.

[58] Christophorou MA, Castelo-Branco G, Halley-Stott RP, Oliveira CS, Loos R, Radzisheuskaya A, Mowen KA, Bertone P, Silva JC, Zernicka-Goetz M (2014). Citrullination regulates pluripotency and histone H1 binding to chromatin. Nature.

[59] Moshkovich N, Ochoa HJ, Tang B, Yang HH, Yang Y, Huang J, Lee MP, Wakefield LM (2020). Peptidylarginine deiminase IV regulates breast cancer stem cells via a novel tumor cell-autonomous suppressor role. Can Res.

[60] Zheng ZQ, Li ZX, Guan JL, Liu X, Li JY, Chen Y, Lin L, Kou J, Lv JW, Zhang LL (2020). Long non-coding RNA TINCR-mediated regulation of acetyl-CoA metabolism promotes nasopharyngeal carcinoma progression and chemoresistance. Cancer Res.

[61] Zhang X, Liu X, Zhang M, Li T, Muth A, Thompson PR, Coonrod SA, Zhang X (2016). Peptidylarginine deiminase 1-catalyzed histone citrullination is essential for early embryo development. Sci Rep.

[62] Hu E, Du H, Shang S, Zhang Y, Lu X (2020). Beta-hydroxybutyrate enhances BDNF expression by increasing H3K4me3 and decreasing H2AK119ub in hippocampal neurons. Front Neurosci.

[63] Kasinath V, Beck C, Sauer P, Poepsel S, Kosmatka J, Faini M, Toso D, Aebersold R, Nogales E (2021). JARID2 and AEBP2 regulate PRC2 in the presence of H2AK119ub1 and other histone modifications. Science.

[64] Burr ML, Sparbier CE, Chan KL, Chan YC, Kersbergen A, Lam EYN, Azidis-Yates E, Vassiliadis D, Bell CC, Gilan O (2019). An evolutionarily conserved function of polycomb silences the MHC class I antigen presentation pathway and enables immune evasion in cancer. Cancer Cell.

[65] Hoshii T, Cifani P, Feng Z, Huang CH, Koche R, Chen CW, Delaney CD, Lowe SW, Kentsis A, Armstrong SA (2018). A non-catalytic function of SETD1A regulates cyclin K and the DNA damage response. Cell.

[66] Ogawa S, Fukuda A, Matsumoto Y, Hanyu Y, Sono M, Fukunaga Y, Masuda T, Araki O, Nagao M, Yoshikawa T (2020). SETDB1 inhibits p53-mediated apoptosis and is required for formation of pancreatic ductal adenocarcinomas in mice. Gastroenterology.

[67] Xie H, Zhao J, Wan J, Zhao J, Wang Q, Yang X, Yang W, Lin P, Yu X (2020). Long non-coding RNA AC245100.4 promotes prostate cancer tumorigenesis via the microRNA-145-5p/RBBP5 axis. Oncol Rep.

[68] Kitai Y, Iwakami M, Saitoh K, Togi S, Isayama S, Sekine Y, Muromoto R, Kashiwakura JI, Yoshimura A, Oritani K (2017). STAP-2 protein promotes prostate cancer growth by enhancing epidermal growth factor receptor stabilization. J Biol Chem.

[69] Laurin M, Huber J, Pelletier A, Houalla T, Park M, Fukui Y, Haibe-Kains B, Muller WJ, Côté JF (2013). Rac-specific guanine nucleotide exchange factor DOCK1 is a critical regulator of HER2-mediated breast cancer metastasis. Proc Natl Acad Sci USA.

[70] Tinnikov AA, Yeung KT, Das S, Samuels HH (2009). Identification of a novel pathway that selectively modulates apoptosis of breast cancer cells. Can Res.

[71] Lee JY, Joo HS, Choi HJ, Jin S, Kim HY, Jeong GY, An HW, Park MK, Lee SE, Kim WS (2019). Role of MEL-18 amplification in Anti-HER2 therapy of breast cancer. J Natl Cancer Inst.

[72] Zhang XW, Sheng YP, Li Q, Qin W, Lu YW, Cheng YF, Liu BY, Zhang FC, Li J, Dimri GP (2010). BMI1 and Mel-18 oppositely regulate carcinogenesis and progression of gastric cancer. Mol Cancer.

[73] Hong ZF, Zhang WQ, Wang SJ, Li SY, Shang J, Liu F, Shen DY (2020). Upregulation of DPY30 promotes cell proliferation and predicts a poor prognosis in cholangiocarcinoma. Biomed Pharmacother = Biomedecine & Pharmacotherapie.

[74] He M, Jin Q, Chen C, Liu Y, Ye X, Jiang Y, Ji F, Qian H, Gan D, Yue S (2019). The miR-186-3p/EREG axis orchestrates tamoxifen resistance and aerobic glycolysis in breast cancer cells. Oncogene.

[75] Xiong S, Tu H, Kollareddy M, Pant V, Li Q, Zhang Y, Jackson JG, Suh YA, Elizondo-Fraire AC, Yang P (2014). Pla2g16 phospholipase mediates gain-of-function activities of mutant p53. Proc Natl Acad Sci USA.

[76] Fang Y, Han X, Li J, Kuang T, Lou W (2020). HEATR1 deficiency promotes chemoresistance via upregulating ZNF185 and downregulating SMAD4 in pancreatic cancer. J Oncol.

[77] Jin H, Yang L, Wang L, Yang Z, Zhan Q, Tao Y, Zou Q, Tang Y, Xian J, Zhang S (2018). INPP4B promotes cell survival via SGK3 activation in NPM1-mutated leukemia. J Exp Clin Cancer Res.

[78] Khalid M, Idichi T, Seki N, Wada M, Yamada Y, Fukuhisa H, Toda H, Kita Y, Kawasaki Y, Tanoue K (2019). Gene regulation by antitumor miR-204-5p in pancreatic ductal adenocarcinoma: the clinical significance of direct RACGAP1 regulation. Cancers.

[79] Li N, Fassl A, Chick J, Inuzuka H, Li X, Mansour MR, Liu L, Wang H, King B, Shaik S (2014). Cyclin C is a haploinsufficient tumour suppressor. Nat Cell Biol.

[80] Togami K, Pastika T, Stephansky J, Ghandi M, Christie AL, Jones KL, Johnson CA, Lindsay RW, Brooks CL, Letai A (2019). DNA methyltransferase inhibition overcomes diphthamide pathway deficiencies underlying CD123-targeted treatment resistance. J Clin Investig.

[81] Pedersen MG, Sherman A (2009). Newcomer insulin secretory granules as a highly calcium-sensitive pool. Proc Natl Acad Sci USA.

[82] Shibasaki T, Takahashi H, Miki T, Sunaga Y, Matsumura K, Yamanaka M, Zhang C, Tamamoto A, Satoh T, Miyazaki J (2007). Essential role of Epac2/Rap1 signaling in regulation of insulin granule dynamics by cAMP. Proc Natl Acad Sci USA.

[83] Capozzi ME, DiMarchi RD, Tschöp MH, Finan B, Campbell JE (2018). Targeting the incretin/glucagon system with triagonists to treat diabetes. Endocr Rev.

[84] Rahn S, Zimmermann V, Viol F, Knaack H, Stemmer K, Peters L, Lenk L, Ungefroren H, Saur D, Schäfer H (2018). Diabetes as risk factor for pancreatic cancer: hyperglycemia promotes epithelial–mesenchymal-transition and stem cell properties in pancreatic ductal epithelial cells. Cancer Lett.

[85] Duan Q, Li H, Gao C, Zhao H, Wu S, Wu H, Wang C, Shen Q, Yin T (2019). High glucose promotes pancreatic cancer cells to escape from immune surveillance via AMPK-Bmi1-GATA2-MICA/B pathway. J Exp Clin Cancer Res.

[86] Hart PA, Bellin MD, Andersen DK, Bradley D, Cruz-Monserrate Z, Forsmark CE, Goodarzi MO, Habtezion A, Korc M, Kudva YC (2016). Type 3c (pancreatogenic) diabetes mellitus secondary to chronic pancreatitis and pancreatic cancer. Lancet Gastroenterol Hepatol.

[87] Zhang AMY, Magrill J, de Winter TJJ, Hu X, Skovsø S, Schaeffer DF, Kopp JL, Johnson JD (2019). Endogenous hyperinsulinemia contributes to pancreatic cancer development. Cell Metab.

[88] Mutgan AC, Besikcioglu HE, Wang S, Friess H, Ceyhan GO, Demir IE (2018). Insulin/IGF-driven cancer cell-stroma crosstalk as a novel therapeutic target in pancreatic cancer. Mol Cancer.

[89] Pannala R, Leirness JB, Bamlet WR, Basu A, Petersen GM, Chari ST (2008). Prevalence and clinical profile of pancreatic cancer-associated diabetes mellitus. Gastroenterology.

[90] Raghavan SR, Ballehaninna UK, Chamberlain RS (2013). The impact of perioperative blood glucose levels on pancreatic cancer prognosis and surgical outcomes: an evidence-based review. Pancreas.

[91] Mazur PK, Herner A, Mello SS, Wirth M, Hausmann S, Sánchez-Rivera FJ, Lofgren SM, Kuschma T, Hahn SA, Vangala D (2015). Combined inhibition of BET family proteins and histone deacetylases as a potential epigenetics-based therapy for pancreatic ductal adenocarcinoma. Nat Med.

[92] Mishra VK, Wegwitz F, Kosinsky RL, Sen M, Baumgartner R, Wulff T, Siveke JT, Schildhaus HU, Najafova Z, Kari V (2017). Histone deacetylase class-I inhibition promotes epithelial gene expression in pancreatic cancer cells in a BRD4- and MYC-dependent manner. Nucleic Acids Res.

[93] He S, Dong G, Li Y, Wu S, Wang W, Sheng C (2020). Potent dual BET/HDAC inhibitors for efficient treatment of pancreatic cancer. Angew Chem Int Ed Engl.

[94] Hessmann E, Johnsen SA, Siveke JT, Ellenrieder V (2017). Epigenetic treatment of pancreatic cancer: is there a therapeutic perspective on the horizon?. Gut.

[95] Kim SS, Xu S, Cui J, Poddar S, Le TM, Hayrapetyan H, Li L, Wu N, Moore AM, Zhou L (2020). Histone deacetylase inhibition is synthetically lethal with arginine deprivation in pancreatic cancers with low argininosuccinate synthetase 1 expression. Theranostics.

[96] Xue N, Lai F, Du T, Ji M, Liu D, Yan C, Zhang S, Yu X, Jin J, Chen X (2019). Chaperone-mediated autophagy degradation of IGF-1Rβ induced by NVP-AUY922 in pancreatic cancer. Cell Mol Life Sci.

