# Supplementary figures and images for: Construction of a prognostic model with histone modification-related genes and identification of potential drugs in pancreatic cancer
Source: Cancer Cell Int. 2021 Jun 5;21:291. doi: 10.1186/s12935-021-01928-6 (PMC8178883; doi:10.1186/s12935-021-01928-6)

**A**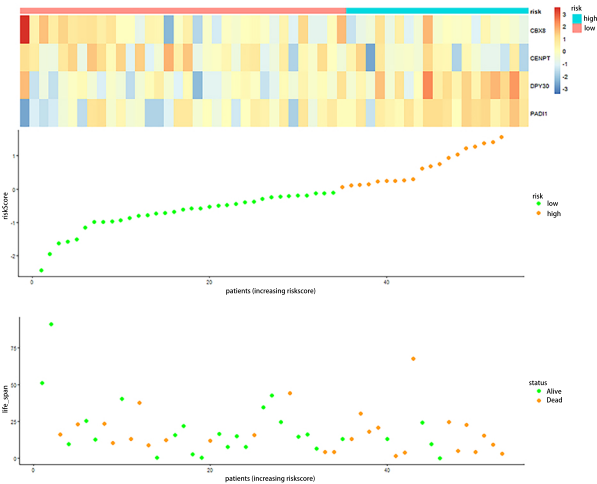**B**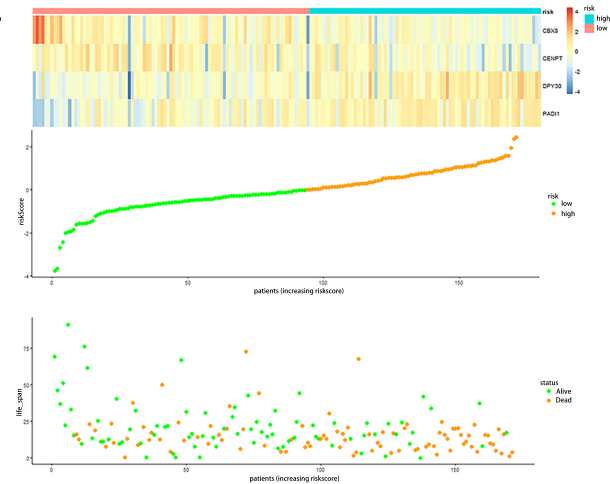**C**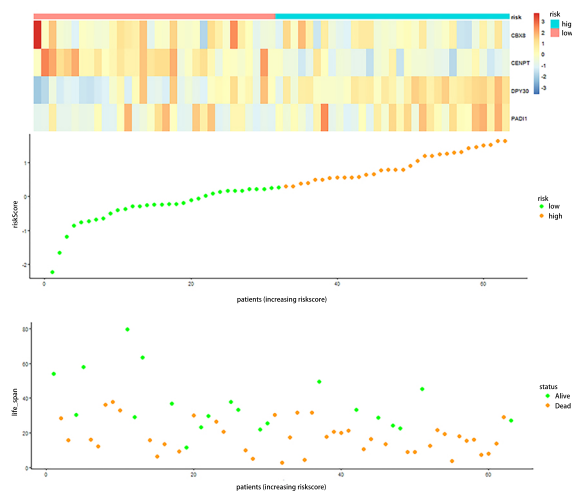

Supplement: Supplementary file 5 — Additional file 5: Figure S1. Further validation of the risk signature for the survival prediction in testing set, the entire TCGA set and the GSE57495 set. (A–C) Heatmap of the four genes expression, the risk scores distribution and survival status of the patients in the testing set, entire TCGA set and GSE57495 set. [file 12935_2021_1928_MOESM5_ESM.pdf]

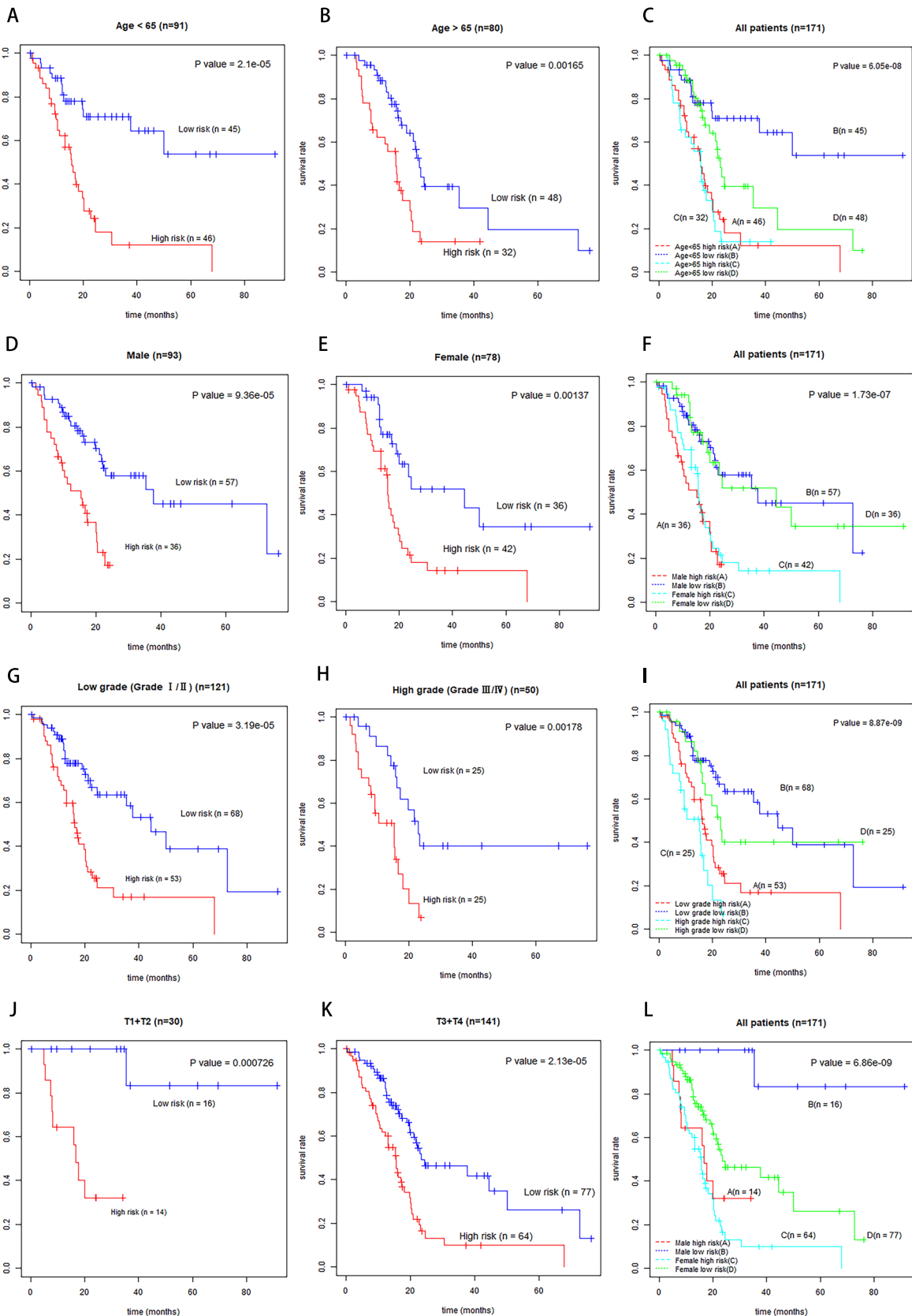

Supplement: Supplementary file 6 — Additional file 6: Figure S2. Stratification analyses of all patients using the risk signature. (A–C) The Kaplan–Meier plot of the younger stratum (age ≤ 65, n = 91), older stratum (age > 65, n = 80) and entire patients with PC (n = 171). (D–F) The Kaplan–Meier plot of the male stratum (n = 93), female stratum n = 78) and entire patients with PC (n = 171). (G–I) The Kaplan–Meier plot of the Grade I/II stratum (n = 121), Grade III/IV stratum (n = 50) and entire patients with PC (n = 171). (J–L) The Kaplan–Meier plot of the T1 + T2 stratum (n = 30), T3 + T4 stratum (n = 141) and entire patients with PC (n = 171). [file 12935_2021_1928_MOESM6_ESM.pdf]

A

## Univariable Cox Regression Analysis

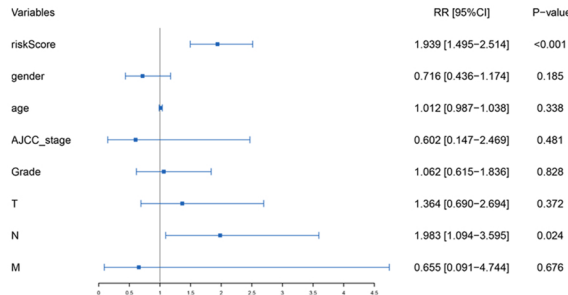

B

## Multivariable Cox Regression Analysis

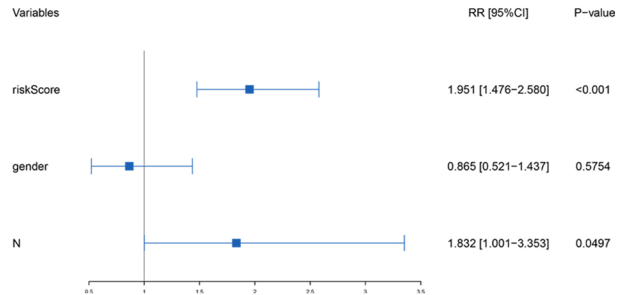

C

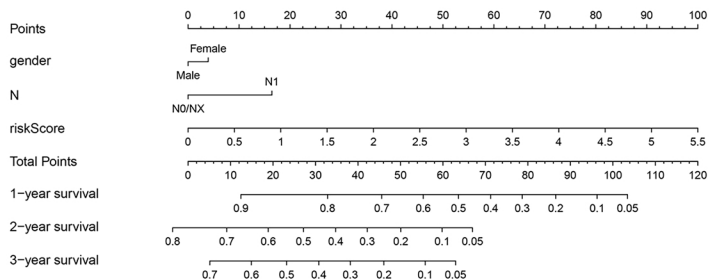

Supplement: Supplementary file 7 — Additional file 7: Figure S3. Construction of a nomogram for predicting 1-, 2- and 3-year survival rate of PC. (A) Forrest plot of univariate Cox regression analysis in training set. (B) Forrest plot of multivariate Cox regression analysis in in training set. (C) Nomogram integrating four histone modification gene-based risk score, gender and N stage. [file 12935_2021_1928_MOESM7_ESM.pdf]

**A**

Training set

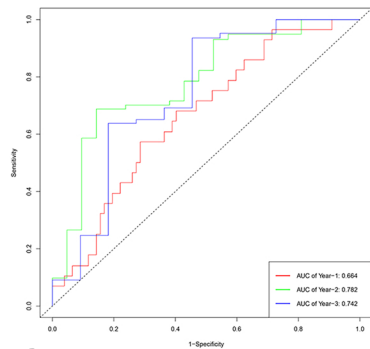**B**

Testing set

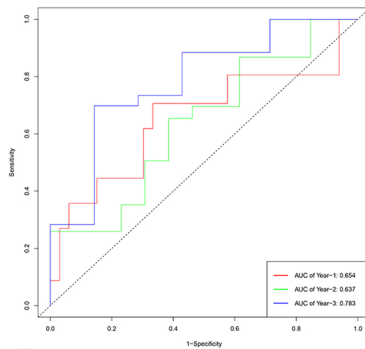**C**

Entire set

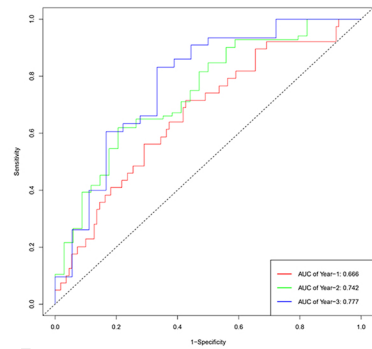**D**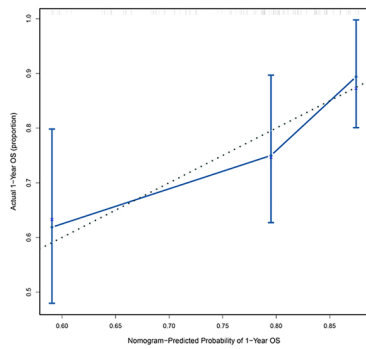**E**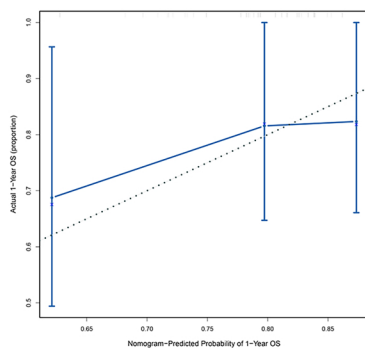**F**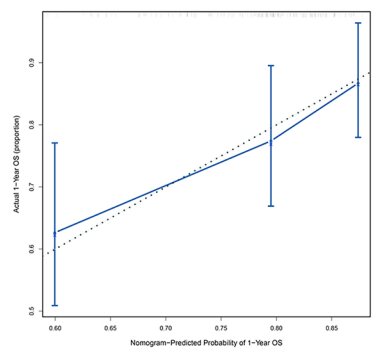**G**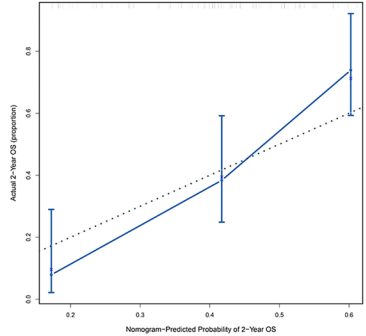**H**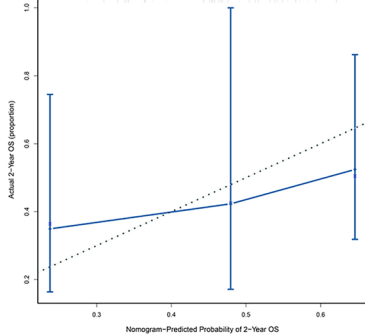**I**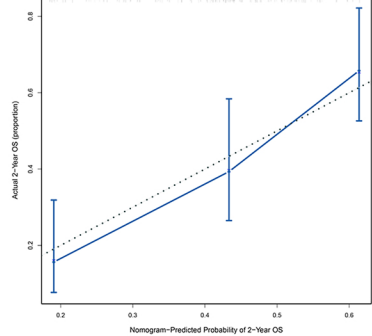**J**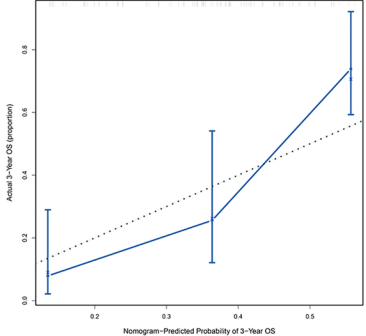**K**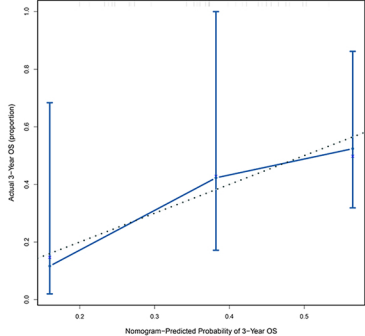**L**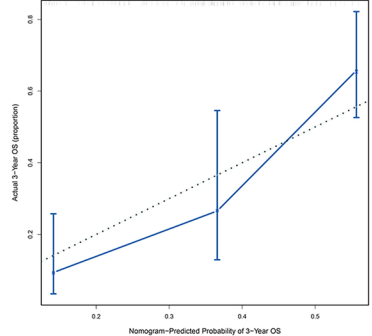

Supplement: Supplementary file 8 — Additional file 8: Figure S4. Validation of the nomogram in training set, testing set and entire set. (A–C) Time-dependent ROC analysis of the risk signature in training set, testing set and entire set. (D–L) The calibration plot of the nomogram for agreement test between 1-, 2- and 3-year OS prediction and actual outcome in the training set, testing set and entire set. [file 12935_2021_1928_MOESM8_ESM.pdf]

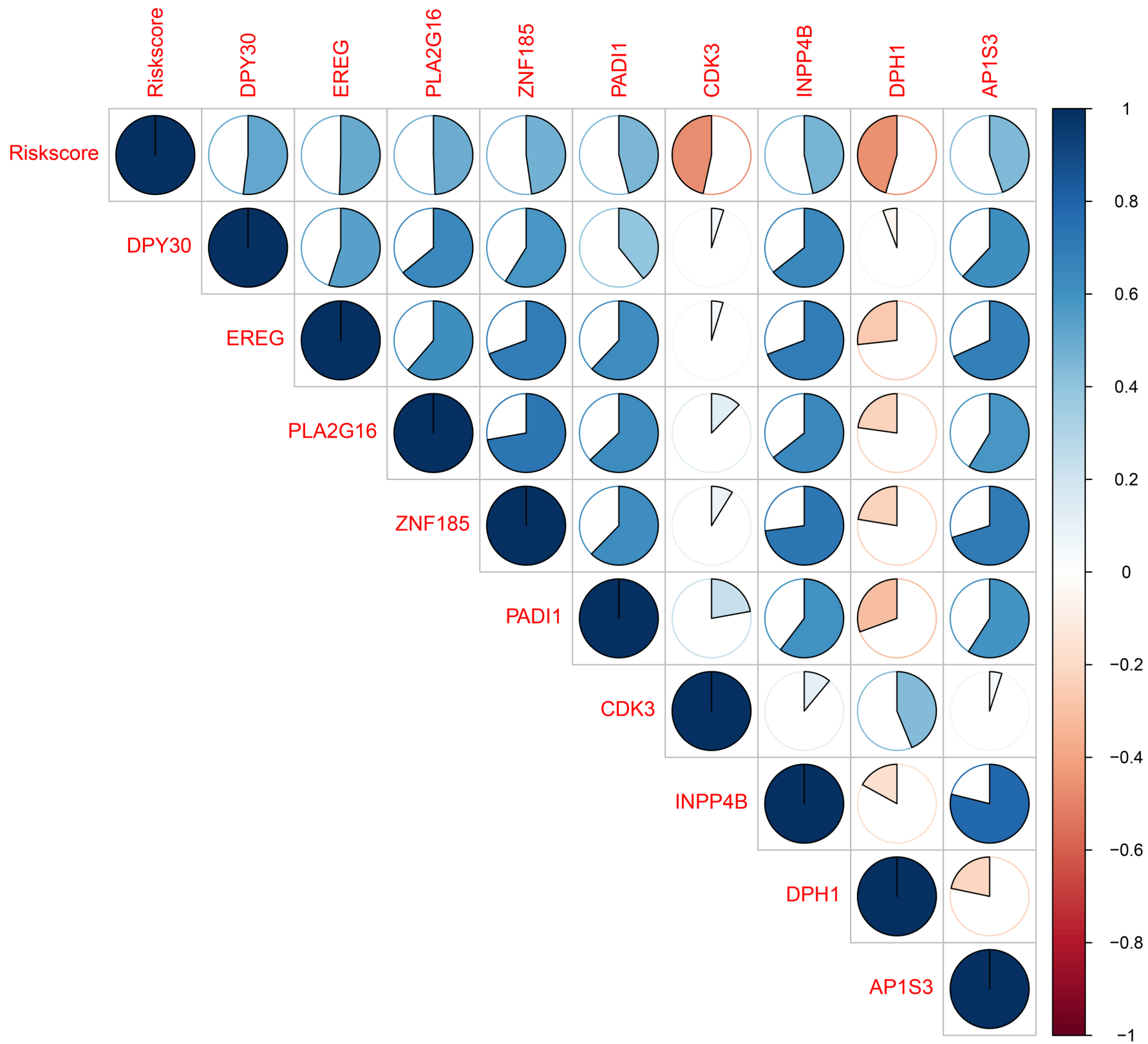

Supplement: Supplementary file 10 — Additional file 10: Figure S6. Correlation between risk score and expression of tumor-related genes through Pearson correlation analysis in TCGA dataset. [file 12935_2021_1928_MOESM10_ESM.pdf]

A

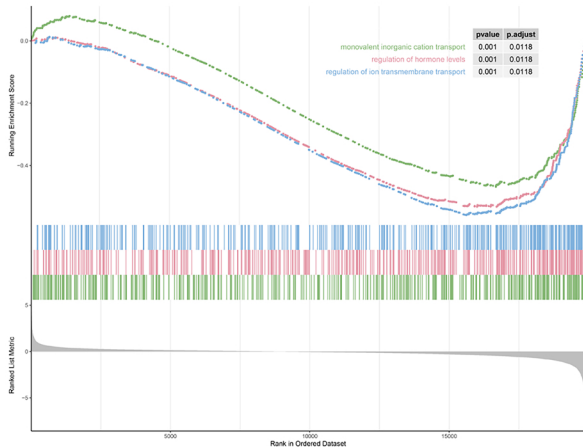

B

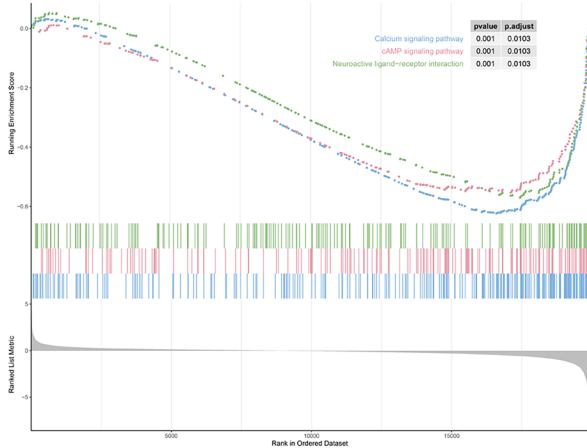

Supplement: Supplementary file 11 — Additional file 11: Figure S7. Gene Set Enrichment Analysis (GSEA) based on all genes between low and high risk groups. The top 3 GO enrichments (A) and KEGG enrichments (B) are displayed on the top respectively. [file 12935_2021_1928_MOESM11_ESM.pdf]

A

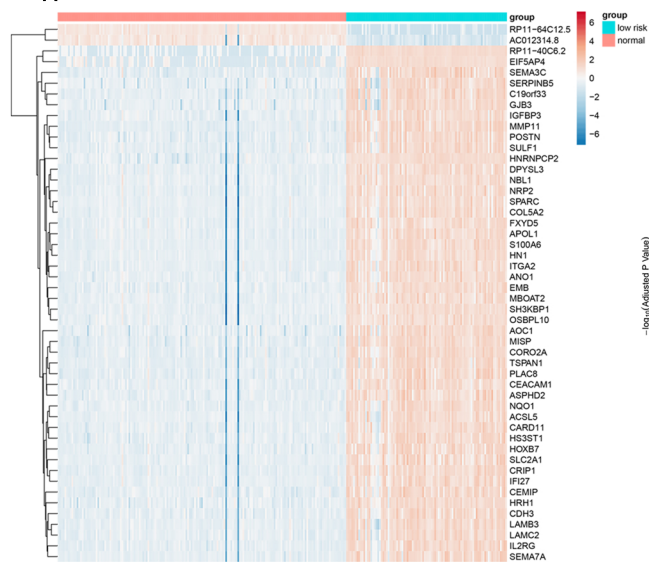

B

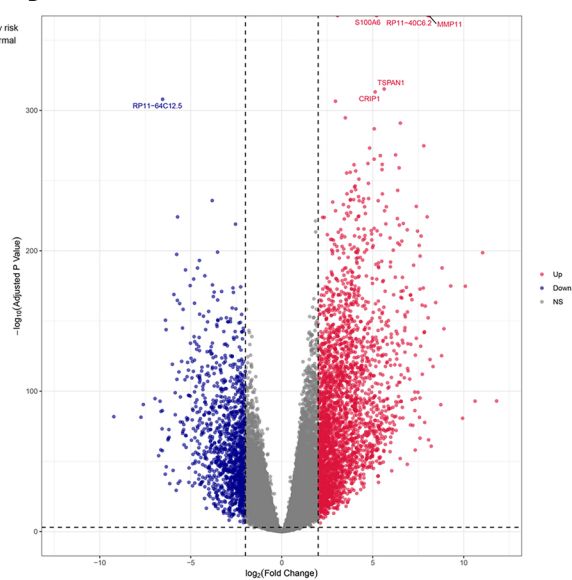

C

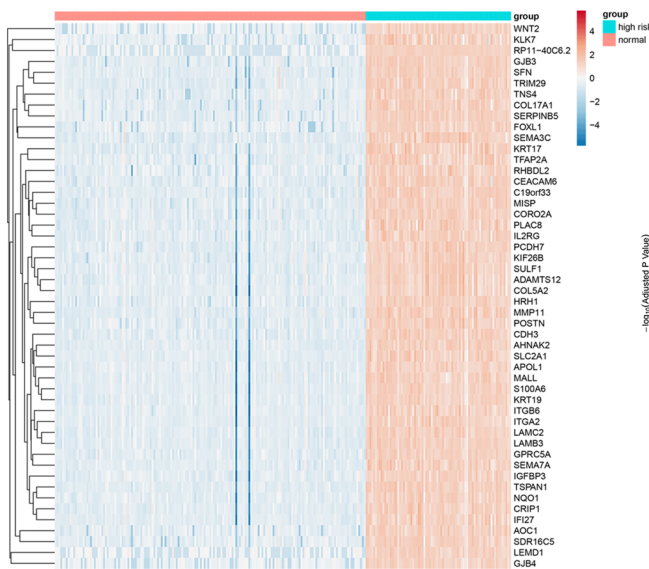

D

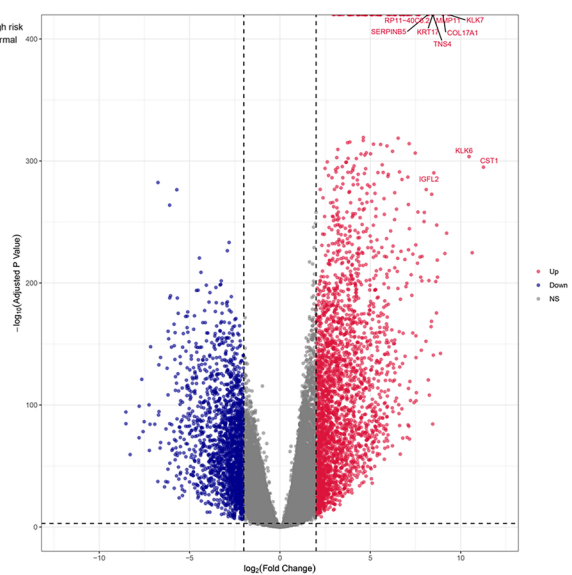

Supplement: Supplementary file 12 — Additional file 12: Figure S8. Differential gene expression in the high- and low-risk group compared with the normal group. (A) Heatmap of DEGs (Top50) between the low-risk pancreatic cancer group and the normal group. (B) Volcanic map of the DEGs between the low-risk pancreatic cancer group and the normal group. (C) Heatmap of DEGs (Top50) between the high-risk pancreatic cancer group and the normal group. (D) Volcanic map of the DEGs between the high-risk pancreatic cancer group and the normal group [file 12935_2021_1928_MOESM12_ESM.pdf]
