# Supplementary material for: Construction of a prognostic model with histone modification-related genes and identification of potential drugs in pancreatic cancer
Source: Cancer Cell Int. 2021 Jun 5;21:291. doi: 10.1186/s12935-021-01928-6 (PMC8178883; doi:10.1186/s12935-021-01928-6)
Supplement: Supplementary file 9 — Additional file 9: Figure S5. The mutations status and mechanisms of function of DPY30, CBX8, CENPT and PADI1. (A) Mutation information of DPY30, CBX8, CENPT and PADI1 from the cBioPortal database. (B–E) Interaction of DPY30, CBX8, CENPT and PADI1 with other proteins obtained from STRING database and validation in the TCGA entire set. [file 12935_2021_1928_MOESM9_ESM.pdf]

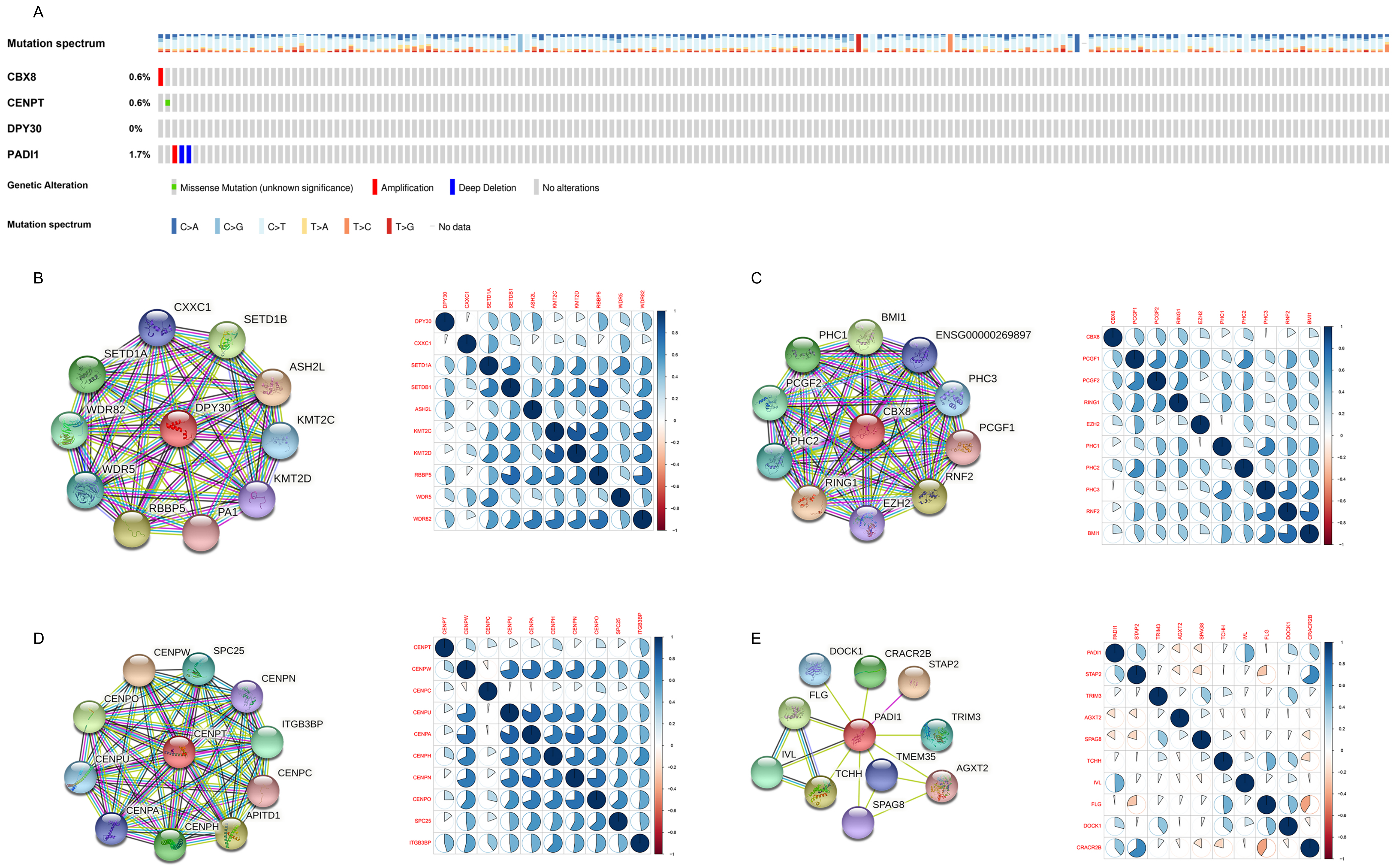
A
Mutation spectrum
CBX8 0.6%
CENPT 0.6%
DPY30 0%
PADI1 1.7%
Genetic Alteration
Missense Mutation (unknown significance)
Amplification
Deep Deletion
No alterations
Mutation spectrum
C>A
C>G
C>T
T>A
T>C
T>G
No data
B
CXXC1
SETD1B
SETD1A
ASH2L
WDR82
DPY30
KMT2C
WDR5
KMT2D
RBBP5
PA1
DPY30 CXXC1 SETD1A SETD1B ASH2L KMT2C KMT2D RBBP5 WDR5 WDR82
C
BMI1
PHC1
ENSG00000269897
PHC3
PCGF2
CBX8
PCGF1
PHC2
RING1
RNF2
EZH2
CBX8 PCGF1 PCGF2 RING1 EZH2 PHC1 PHC2 PHC3 RNF2 BMI1
D
CENPW
SPC25
CENPN
CENPO
ITGB3BP
CENPT
CENPU
CENPC
CENPA
APITD1
CENPH
CENPT CENPW CENPC CENPU CENPA CENPH CENPN CENPO SPC25 ITGB3BP
E
DOCK1
CRACR2B
STAP2
FLG
PADI1
TRIM3
IVL
TMEM35
AGXT2
TCHH
SPAG8
PADI1 STAP2 TRIM3 AGXT2 SPAG8 TCHH IVL FLG DOCK1 CRACR2B
1
0.8
0.6
0.4
0.2
0
-0.2
-0.4
-0.6
-0.8
-1
